# Inhibitory Effect of Chlorogenic Acid Analogues Comprising Pyridine and Pyrimidine on α-MSH-Stimulated Melanogenesis and Stability of Acyl Analogues in Methanol

**DOI:** 10.3390/ph14111176

**Published:** 2021-11-17

**Authors:** Jaeuk Sim, Srinu Lanka, Jeong-Woong Jo, Chhabi Lal Chaudhary, Manjunatha Vishwanath, Chan-Hyun Jung, Young-Hee Lee, Eun-Yeong Kim, Young-Soo Kim, Soon-Sil Hyun, Hee-Soon Lee, Kiho Lee, Seung-Yong Seo, Mayavan Viji, Jae-Kyung Jung

**Affiliations:** 1 College of Pharmacy and Medicinal Research Center (MRC), Chungbuk National University, Cheongju 28160, Korea; simprog@naver.com (J.S.); lsrinu23@gmail.com (S.L.); cjw1881@naver.com (J.-W.J.); chhabichaudhary319@gmail.com (C.L.C.); 16vishwanath@gmail.com (M.V.); cksgus9823@naver.com (C.-H.J.); yhlee91@daum.net (Y.-H.L.); youngsoo@chungbuk.ac.kr (Y.-S.K.); shyun@chungbuk.ac.kr (S.-S.H.); medchem@chungbuk.ac.kr (H.-S.L.); 2 Samjin Central Research Institute, Samjin Pharma Co., Ltd., Cheongju 28158, Korea; 3 College of Pharmacy, Korea University, Sejong 30019, Korea; y215_@naver.com (E.-Y.K.); kiholee@korea.ac.kr (K.L.); 4 Gachon Institute of Pharmaceutical Sciences, College of Pharmacy, Gachon University, Incheon 21936, Korea; syseo@gachon.ac.kr

**Keywords:** chlorogenic acid, α-MSH-stimulated melanogenesis, pyridine, pyrimidine, B16 melanoma cells

## Abstract

In continuation of studies for α-MSH stimulated melanogenesis inhibitors, we have evaluated the design, synthesis, and activity of a new series of chlorogenic acid (CGA) analogues comprising pyridine, pyrimidine, and diacyl derivatives. Among nineteen synthesized compounds, most of them (fifteen) exhibited better inhibitions of melanin formation in B16 melanoma cells. The results illustrated that a pyridine analogue **6f** and a diacyl derivative **13a** of CGA showed superior inhibition profiles (IC_50_: 2.5 ± 0.7 μM and 1.1 ± 0.1 μM, respectively) of *α*-MSH activities than positive controls, kojic acid and arbutin (IC_50_: 54 ± 1.5 μM and 380 ± 9.5 μM, respectively). The SAR studies showed that both –CF_3_ and –Cl groups exhibited better inhibition at the *meta* position on benzylamine than their *ortho* and *para* positions. In addition, the stability of diacyl analogues of CGA in methanol monitored by HPLC for 28 days indicated the steric bulkiness of acyl substituents as a key factor in their stability.

## 1. Introduction

Melanin, produced by the melanocytes through the complex melanogenesis process, plays a significant role in determining the color of human skin, eye, and hair [1,2,3,4,5]. Melanocytes are known to be stimulated by various factors including UV radiation, melanocyte-stimulating hormone (α-MSH), a phosphodiesterase inhibitor, such as theophylline. Mainly three tyrosinase family enzymes are involved in the regulation of melanogenesis in mammalians such as tyrosinase and two tyrosinase-related proteins, TYRP1 and TYRP2. Among these, tyrosinase (EC.1.14.18.1) is the main rate-limiting enzyme that controls the biosynthesis of melanin production by the conversion of L-tyrosine to dopaquinone via L-dopa using monophenolase and diphenolase activities [6,7,8,9]. However, an excess secretion of melanin from melanocytes due to prolonged exposure to sunlight leads to dermatologic disorders such as melasma [10], freckles [11], post-inflammatory melanoderma [12], solar lentigines [13], vitiligo [14], and even cancer [15]. Moreover, a wide number of research revealed that many melanogenesis disorders have been linked to neurodegenerative diseases, including Parkinson’s, Alzheimer’s, and Huntington’s diseases as well [16,17,18]. Therefore, the skincare industry is continuously growing around the globe in recent days. Approximately ≈15% of the world’s population are consuming skincare and cosmetic products; among them, Asia lies on the highest rank [19].

In this regard, chemists have been paying more attention to make novel skincare products to reduce the effect of pigmentation such as skin-whitening agents with no side effects [20,21,22,23,24,25,26,27,28,29]. Several small heterocyclic molecules are playing a vital role in the field of medicinal chemistry. A wide variety of synthetic agents such as kojic acid derivatives [30,31,32], thiosemicarbazones [28,33], Morita–Baylis–Hillman adducts [34], quinazolinone benzamides [35,36], and cinnamic acid derivatives [37,38] are reported for the treatment of hyperpigmentation disorders. Pyridine is a common structural motif that is ubiquitous in several pharmaceuticals and natural products including vitamins and alkaloids [39,40,41,42]. Similarly, pyridazine, a 1,3-diazine six membered molecule, has also been received important attention in medicinal chemistry because of its range of biological properties such as antihypertensive, anti-cancer, anti-HIV, and so on [43,44]. Moreover, a few scaffolds of both pyridines [45,46,47] and pyrimidines [48,49,50] are highly effective against the melanogenesis process. Chlorogenic acid (5-*O*-caffeoylquinic acid, 5-CQA) is a polyphenol ester obtained by the reaction between *trans*-cinnamic acid and quinic acid [51,52,53]. It is predominantly found in beverages and coffee drinks. In addition, several vegetables and fruits such as eggplant, artichoke, sweet potato, tomato, oilseeds, apples, pears, blueberries, and peanuts are also observed as rich sources [54,55,56]. A number of research studies revealed that CGA was used to improve several disorders including obesity and diabetes in traditional medicinal practice. In addition, it exhibits numerous biological activities such as anti-inflammatory, antioxidant, anti-cancer, anti-hypertensive, antimicrobial, anti-obesity, hypolipidemic, liver protective, etc. [57,58,59]. Growing evidence recognized that CGA significantly inhibits the tumor growth in various cancer cell lines. For example, Anqi Zeng et al. and Sapio Luigi et al. have reported the anti-cancer activity of CGA by induction of the apoptosis pathway in breast cancer and osteosarcoma cell lines [60,61]. Likewise, Alessia Salzillo et al. highlighted its synergistic effect in the inhibition of growth and proliferation of osteosarcoma cells potentially via an apoptosis mechanism when used together with doxorubicin [62].

**Figure 1:**
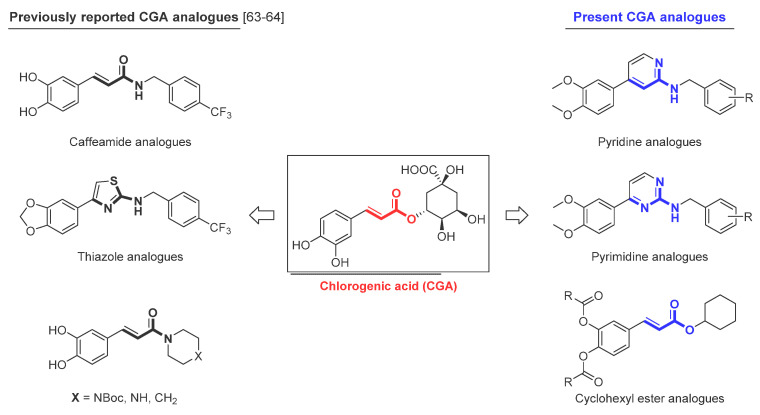
Design strategy for new α-MSH-induced melanogenesis inhibitors.

Considering the importance of skincare products, as a part of our continuous work in the way to the preparation of potent CGA analogues targeting α-MSH-stimulated melanogenesis inhibition [63,64], we have reported the design and synthesis of dimethoxy phenyl analogues of CGA comprising of pyridine, pyrimidine, and cyclohexyl caffeamide skeletons (Figure 1) along with their inhibitory activities against melanin formation. In addition, the stability of diacyl analogues of cyclohexyl ester (i.e., diacyl caffeates) were examined to see the effect of acyl substituents on their stability profiles.

## 2. Results

### 2.1. Chemistry

**Scheme 1:**
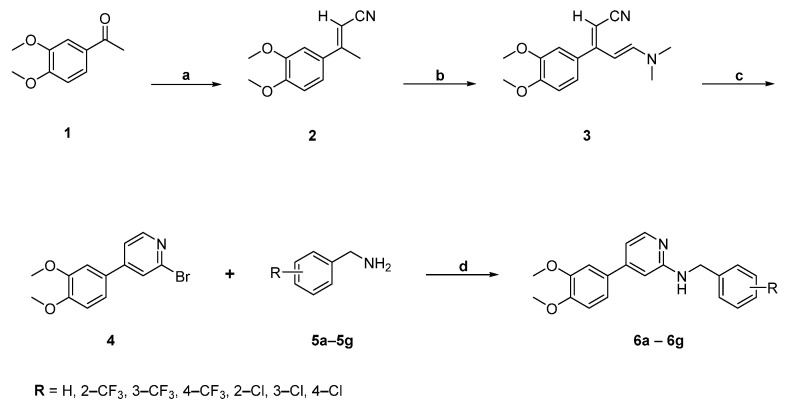
Synthesis of pyridine analogues **6a**–**g** of CGA: Reagents and conditions: (**a**) diethyl cyanomethylphosphonate, NaH, THF, 0 °C; (**b**) DMF-DMA, NH_4_OAc, DMSO, 120 °C; (**c**) AcBr, EA, H_2_O, rt, 53%; (**d**) CsOAc, Cu, DMSO, 90 °C, 20–57%.

Several pyridine-containing chlorogenic acid analogues **6a**–**g** were prepared, as shown in Scheme 1. The starting material *α*,*β*-unsaturated nitrile **2** was obtained by the Horner–Wadsworth–Emmons (HWE) olefination of 3′,4′-dimethoxy acetophenone **1** with diethyl cyanomethyl phosphonate. The *α*,*β*-unsaturated nitrile was treated with DMF-DMA and NH_4_OAc in DMSO at 120 °C yielded enamino nitrile intermediate **3**, which was further reacted with an excess of acetyl bromide in ethyl acetate and H_2_O at 0 °C to form the required bromopyridine intermediate **4** [65]. Furthermore, compound **4** was treated with various type of benzyl amines **5a**–**g** comprising –CF_3_ and –Cl groups on the benzene ring in the presence of CsOAc and Cu at 90 °C in DMSO, which yielded desired pyridine ring-containing analogues **6a**–**g**.

**Scheme 2:**
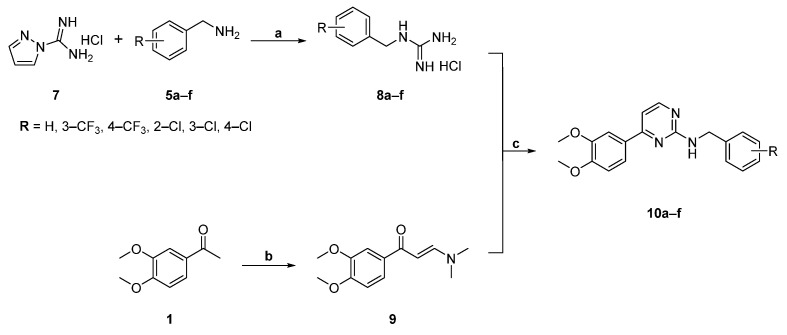
Synthesis of pyrimidine analogues **10a**–**f** of CGA: Reagents and conditions: (**a**) Et_3_N, MeCN, 60 °C, 53–73%; (**b**) DMF-DMA, reflux, 87%; (**c**) K_2_CO_3_, EtOH, reflux, 7–44%.

Various pyrimidine analogues **10a**–**f** were synthesized using the method shown in Scheme 2. Initially, benzyl guanidine intermediates **8a**–**f** [66] were obtained by treating **7** [67] with different types of benzyl amines **5a**–**f** bearing –CF_3_ and –Cl groups. Then, (*E*)-1-(3,4-dimethoxy phenyl)-3-(dimethylamino) prop-2-en-1-one intermediate **9** was obtained by reacting acetophenone **1** with DMF-DMA under a reflux condition. Lastly, both the intermediates **8a**–**f** and **9** were coupled under basic condition at refluxing temperature to afford the target compounds **10a**–**f**.

**Scheme 3:**
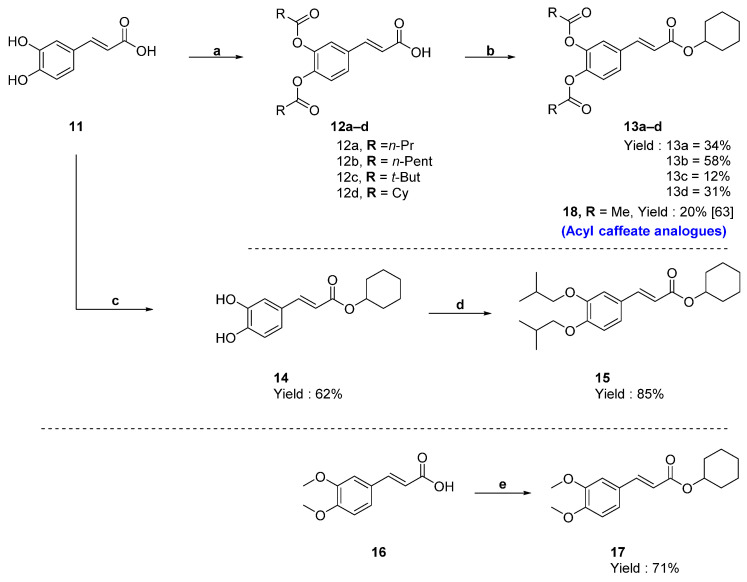
Synthesis of cyclohexyl ester analogues of CGA: Reagents and conditions; (**a**) DMAP, pyridine, acid chloride, rt; (**b**) (i) SOCl_2_, toluene, 100 °C, N_2_; (ii) DMAP, cyclohexanol, CH_2_Cl_2_, rt, N_2_, 12–58%; (**c**) H_2_SO_4_, cyclohexanol, reflux, 62%; (**d**) isobutyl bromide, K_2_CO_3_, DMF, 70 °C, 85%; (**e**) (i) SOCl_2_, toluene, rt; (ii) DMAP, cyclohexanol, CH_2_Cl_2_, rt, 71%.

Furthermore, we approached the synthesis of substituted diacyl **13a**–**d** and dialkyl CGA cyclohexyl ester analogues **15**, **17** as portrayed in Scheme 3. Caffeic acid **11** was *O*-acylated with acid halide in pyridine in the presence of DMAP catalyst to yield corresponding *O*-acylated analogues **12a**–**d**. The obtained analogues **12a**–**d** were further reacted with thionyl chloride in toluene under N_2_ atmosphere at 100 °C, which was followed by esterification using cyclohexanol catalyzed by DMAP at rt, which afforded **13a**–**d**. Similarly, compound **15** was obtained by treating caffeic acid **11** with cyclohexanol in refluxing sulfuric acid affording cyclohexyl ester precursor **14**, which was followed by *O*-alkylation of **14** with isobutyl bromide in basic condition. On the other hand, the compound **17**, a dimethoxy caffeic acid cyclohexyl ester analogue, was prepared by reacting dimethoxy caffeic acid **16** with thionyl chloride in refluxing toluene followed by esterification with cyclohexanol at rt, affording good yield.

### 2.2. Biological Results

Pyridine (**6a**–**g**), pyrimidine (**10a**–**f**), and cyclohexyl ester (**13a**–**d**, **15** and **17**) analogues of CGA in hand, we evaluated their inhibitory activity of melanin formation in B16 melanoma cells under stimulus of α-MSH, and the IC_50_ values are summarized in Table 1 (see Supplementary Materials). All the newly synthesized analogues exhibited superior inhibition activity compared with arbutine (a positive control, IC_50_: 380.0 ± 9.5 μM); however, few analogues displayed a lower inhibitory effect than kojic acid (another positive control, IC_50_: 54.0 ± 1.5 μM).

All pyridine skeletons containing analogues (Table 1, **6a**–**g**) exhibited good inhibition of melanin formation in B16 melanoma cells compared to two positive controls (i.e., kojic acid). Based on the degree of inhibition ability, compound **6f** (IC_50_: 2.5 ± 0.7 μM) containing –CF_3_ at the benzylic *meta* position was found to be excellent among the pyridine series. However, its *para* and *ortho* position (**6e**, IC_50_: 8.5 ± 0.7 μM and **6g**, IC_50_: 7.5 ± 0.7, respectively) showed slightly diminished inhibitions. The –Cl substitution at the benzylic *para* and *meta* position (**6b**, IC_50_: 4.5 ± 0.7 μM and **6c**, IC_50_: 5.0 ± 1.4, respectively) have similar inhibitions, but its *ortho* position showed somewhat lower inhibition effect (**6d**, IC_50_: 8.5 ± 0.7 μM). It seems that pyridine analogues have better activity with electron-withdrawing substituents, since benzyl (i.e., without an electron-withdrawing group) analogue **6a** was found to be the least active in the series.

Compared to pyridine analogues, the pyrimidine analogues displayed a lower inhibition power of melanin production. Analogues **10c** (IC_50_: 32.0 ± 2.0 μM), **10d** (IC_50_: 40.0 ± 8.0 μM), and **10f** (IC_50_: 20.0 ± 1.0 μM) were found to be better in inhibition ability compared to positive control kojic acid. However, the analogues **10a** without benzyl substitution and **10e** with –CF_3_ at the benzylic *para* position were weaker in inhibition power than kojic acid, displaying more than 100.0 μM IC_50_ values. Although most analogues were better than the positive control, a clear correlation of structure–activity relationship (SAR) in this series was not observed.

Likewise, the activity against α-MSH stimulated melanin formation in B16 melanoma cells by the acyl **13a**–**d**, and alkyl **15**, **17** caffeic acid cyclohexyl ester derivatives were performed. As the result depicted in Table 1, acyl **13a**–**d** showed a highly potent inhibition of α-MSH stimulation compared to kojic acid and most of the pyridine analogues of CGA. All analogues **13a**–**d** exhibited IC_50_ values lower than 3.0 μM concentration. The SAR study indicated that the activity of acyl analogues is marginally diminished in increasing length of carbon chains and steric crowds of acyl substituents. For example, analogue **13a** (IC_50_: 1.0 ± 0.1 μM) with *n*-propyl substitution was found to be the most potent, whereas analogue **13d** (IC_50_: 2.3 ± 0.2 μM) with the largest substituent (i.e., cyclohexane) unit among the series displayed slight inferior potency. Likewise, alkyl (**15** and **17**) analogues of caffeic acid cyclohexyl esters were also poorly active to inhibition of melanin formation (i.e., IC_50_: >100 μM).

Our previous report demonstrated that most CGA analogues displayed a wide margin of safety window to their IC_50_ value of inhibitory effect when cytotoxic effects were measured by the MTT assay method [63,64]. Herein, the results were similar to the previous report for the new analogues where the preliminary assay showed no significant cytotoxic effects in >30 μM concentration except for analogue **6f**. However, their cytotoxic IC_50_ value will be measured in our further works.

**Figure 2:**
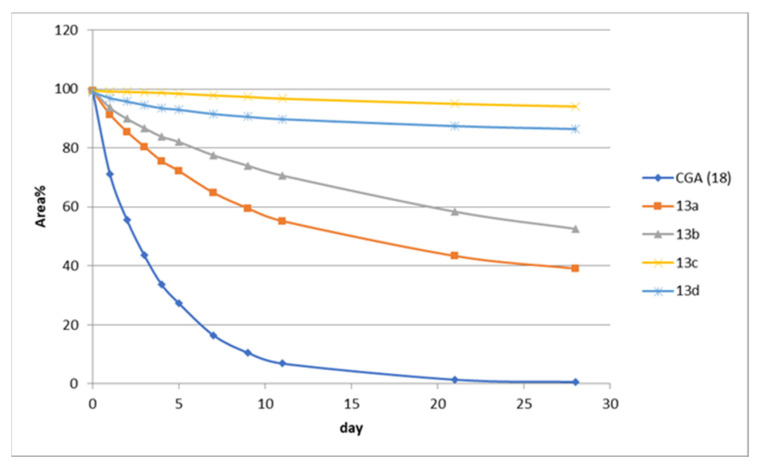
Stability of CGA acyl analogues (Area%) over the time in methanol.

As we continuously utilized caffeamide and caffeate analogues to investigate the inhibition of α-MSH stimulation on B16 melanoma cells, we found diacetyl catechol derivative (**18**, IC_50_: 0.02 ± 1.05) as a highly potent analogue in previous work [63]. However, it was found to be highly unstable upon normal storage condition at room temperature because of the reactive acyl moiety. Since the activity of acyl analogues (**13a**–**13d**) of caffeates are highly promising in the present work as well, we aimed to perform chemical stability testing of synthesized acyl analogues in methanol solution, and their stability profile is depicted in Figure 2 and Table 2. The detailed procedure is given in the Section 4.

As in our previous observation, the caffeate analogue **18** was found to be the least stable compound as it was decomposed by more than 90% within 10 days, and its amount was dropped to less than 1% in 28 days in methanol solution. In this series, cyclohexyl acyl analogue **13d** displayed an excellent stability profile by retaining approximately 87% of its amount in methanol even in 28 days. The substrate having *n*-propyl **13a** and *n*-pentyl **13b** substituents showed the moderate stability profile, as 39% and 52% remained unreacted in solution for 28 days, respectively. The result suggested that the linear alkyl acyl–substituents are highly reactive; thus, they can be decomposed easily upon exposure to nucleophiles such as methanol. However, the installations of sterically bulky substituents improve stability. For instance, analogues **13c** with a bulkier *t*-butyl substituent showed the higher stability rate as 94% remains unreacted after 28 days.

## 3. Discussion

Based on the biological result (Table 1) of the currently synthesized analogues, analogue **6f** (IC_50_: 2.5 ± 0.7 μM) most effectively inhibited the melanin production from B16 melanoma cells against α-MSH stimulation among the pyridine series. If we consider the activity of the benzylic substituents with our previous findings [63,64], the superior activity profile of –CF_3_ at the *meta* position of analogue **6f** parallels with corresponding thiazole (IC_50_: 1.8 ± 0.11 μM) and caffeamide (IC_50_: 1.4 ± 0.36 μM) analogues; however, potency was marginally lower than them. The analogue **6c** (IC_50_: 5.0 ± 1.4 μM) with –Cl at the benzylic *meta* position presented improved inhibition than the equivalent caffeamide (IC_50_: 6.0 ± 0.16 μM) and thiazole (IC_50_: >50 μM) analogues.

In general, analogues in the pyrimidine series are less effective than the corresponding caffeamide, thiazole, and pyridine analogues. Although the pyrimidine series are not as effective as the pyridine series in the inhibition of the melanogenesis process, the effect of –CF_3_ at the benzylic *meta* position has a similar pattern, since **10f** (IC_50_: 20.0 ± 1.0 μM) displayed higher activity than its *ortho* (IC_50_: 40.0 ± 8.0 μM) and *para* (IC_50_: >100.0 μM) derivatives.

By observing the result of our current and previous pyridine/pyrimidine and thiozole analogues of CGA, we found it to be less potent in inhibition activity than parent analogues (i.e., caffeamide and caffeate). Despite the higher inhibition ability of caffeamide and caffeate, the stability of the acyl-substituents on phenol were the most challenging. By substituting with bulkier acyl-substituents, the stability profile was improved in the present work. As we found that the most of our analogues potently block the melanogenesis in B16 melanoma cells with a wide safety profile, thus, the CGA analogous might become a good scaffold for the discovery of anticancer agents and/or cosmeceuticals.

## 4. Materials and Methods

### 4.1. General Information

^1^H NMR spectra were recorded on a Jeol RESONANCE ECZ 400S (400 MHz). Chemical shifts are reported in ppm from tetramethylsilane (TMS) with the solvent resonance resulting from incomplete deuteration as the internal reference (CDCl_3_: 7.26 ppm) or relative to TMS (*δ* 0.0). Data are reported as follows: chemical shift (ppm), multiplicity (s = singlet, d = doublet, t = triplet, q = quartet, br = broad, m = multiplet, dd = doublet of doublet, td = triplet of doublet), coupling constants (Hz), number of protons. ^13^C NMR spectra were recorded on a Jeol RESONANCE ECZ 400S (100 MHz) with complete proton decoupling. Chemical shifts are reported in ppm from tetramethylsilane with the solvent as the internal reference (CDCl_3_: 77.16 ppm). High-resolution mass spectrometry was performed on LCQ Fleet-Thermo Scientifics (Waltham, MA, USA). All reactants or reagents were purchased from Aldrich TCI, alfa aesar, and acros were directly used without further purifications. Silica gel column chromatography was performed with Silica Gel of Kieselgel ^60^ (Merck, KGaA, 64271 Darmstadt, Germany).

### 4.2. General Procedure for the Synthesis of Pyridine Analogues (6**a**–**g**) of CGA

2-Bromo-4-(3,4-dimethoxyphenyl) pyridine **4** (0.13 mmol), cesium acetate (0.05 g, 0.28 mmol), and copper powder (1 mg, 0.013 mmol) was taken in anhydrous DMSO (0.13 mL). Then, benzylamine derivatives **5a**–**5g** (0.19 mmol) were added under nitrogen atmosphere, and the reaction mixture was stirred at 90 °C for 24 h. After the completion of the reaction, the reaction mixture was cooled to room temperature, the precipitate was filtered with ethyl acetate, the organic layer was washed with water, dried over anhydrous MgSO_4_, concentrated under reduced pressure, and purified by column chromatography. All the synthesized compounds were identified by ^1^H NMR, ^13^C NMR and HRMS (see Supplementary Materials).

*N*-Benzyl-4-(3,4-dimethoxyphenyl)pyridin-2-amine (**6a**)

Yield: 57%, ^1^H NMR (CDCl_3_, 400 MHz): *δ* 8.14 (d, *J* = 5.2 Hz, 1H), 7.26–7.41 (m, 5H), 7.14 (dd, *J* = 8.3, 2.1 Hz, 1H), 7.03 (d, *J* = 2.1 Hz, 1H), 6.92 (d, *J* = 8.4 Hz, 1H), 6.81 (dd, *J* = 5.3, 1.5 Hz, 1H), 6.53 (s, 1H), 4.95 (t, *J* = 5.5 Hz, 1H), 4.58 (d, *J* = 5.7 Hz, 2H), 3.92 (d, *J* = 2.1 Hz, 6H); ^13^C NMR (CDCl_3,_ 100 MHz) *δ* 159.2, 149.8, 149.7, 149.2, 148.6, 139.3, 131.9, 128.7, 127.5, 127.3, 119.4, 111.8, 111.4, 110.0, 104.2, 56.0, 56.0, 46.5; HRMS *m*/*z* [M + H]^+^ calculated for C_20_H_20_N_2_O_2_: 321.1598; Found: 321.1608.

*N*-(4-Chlorobenzyl)-4-(3,4-dimethoxyphenyl)pyridin-2-amine (**6b**)

Yield: 27%, ^1^H NMR (CDCl_3_, 500 MHz): *δ* 8.13 (d, *J* = 5.3 Hz, 1H), 7.30–7.34 (m, 4H), 7.13 (dd, *J* = 8.3, 2.1 Hz, 1H), 7.02 (d, *J* = 2.1 Hz, 1H), 6.93 (d, *J* = 8.3 Hz, 1H), 6.82 (dd, *J* = 5.3, 1.5 Hz, 1H), 6.50 (d, *J* = 0.7 Hz, 1H), 4.97 (t, *J* = 5.6 Hz, 1H), 4.56 (d, *J* = 5.8 Hz, 3H), 3.92 (d, *J* = 1.4 Hz, 6H); ^13^C NMR (CDCl_3,_ 100 MHz) *δ* 159.0, 149.8, 149.7, 149.2, 148.6, 137.9, 132.9, 131.8, 128.8, 119.4, 112.0, 111.4, 109.9, 104.4, 56.0, 56.0, 45.7; HRMS *m*/*z* [M + H]^+^ calculated for C_20_H_19_ClN_2_O_2_: 355.1208; Found: 355.1219.

*N*-(3-Chlorobenzyl)-4-(3,4-dimethoxyphenyl)pyridin-2-amine (**6c**)

Yield: 40%, ^1^H NMR (CDCl_3_, 500 MHz): *δ* 8.13 (d, *J* = 5.3 Hz, 1H), 7.39 (d, *J* = 1.0 Hz, 1H), 7.22–7.27 (m, 2H), 7.13 (dd, *J* = 8.3, 2.1 Hz, 1H), 7.03 (d, *J* = 2.1 Hz, 1H), 6.93 (d, *J* = 8.3 Hz, 1H), 6.82 (dd, *J* = 5.3, 1.5 Hz, 1H), 6.51 (d, *J* = 0.8 Hz, 1H), 4.99 (t, *J* = 5.7 Hz, 1H), 4.57 (d, *J* = 5.9 Hz, 2H), 3.92 (d, *J* = 0.7 Hz, 6H); ^13^C NMR (CDCl_3,_ 100 MHz) *δ* 158.9, 149.8, 149.7, 149.2, 148.6, 141.7, 134.5, 131.8, 129.9, 127.5, 127.4, 125.5, 119.4, 112.0, 111.4, 109.9, 104.4, 56.0, 56.0, 45.8; HRMS *m*/*z* [M + H]^+^ calculated for C_20_H_19_ClN_2_O_2_: 355.1208; Found: 355.1218.

*N*-(2-Chlorobenzyl)-4-(3,4-dimethoxyphenyl)pyridin-2-amine (**6d**)

Yield: 23%, ^1^H NMR (CDCl_3_, 400 MHz): *δ* 8.14 (d, *J* = 5.3 Hz, 1H), 7.46–7.48 (m, 1H), 7.37–7.39 (m, 1H), 7.20–7.23 (m, 2H), 7.14 (dd, *J* = 8.3, 2.1 Hz, 1H), 7.05 (d, *J* = 2.1 Hz, 1H), 6.92 (d, *J* = 9.4 Hz, 1H), 6.81 (dd, *J* = 5.3, 1.5 Hz, 1H), 6.52 (d, *J* = 0.6 Hz, 1H), 5.06 (t, *J* = 6.0 Hz, 1H), 4.68 (d, *J* = 6.2 Hz, 2H), 3.92 (d, *J* = 2.3 Hz, 6H); ^13^C NMR (CDCl_3,_ 100 MHz) *δ* 159.0, 149.8, 149.7, 149.2, 148.6, 136.6, 133.4, 131.8, 129.6, 129.3, 128.5, 127.0, 119.5, 111.9, 111.4, 110.0, 104.3, 56.0, 56.0, 44.0; HRMS *m*/*z* [M + H]^+^ calculated for C_20_H_19_ClN_2_O_2_: 355.1208; Found: 355.1222.

4-(3,4-Dimethoxyphenyl)-*N*-(4-(trifluoromethyl)benzyl)pyridin-2-amine (**6e**)

Yield: 21%, ^1^H NMR (CDCl_3_, 500 MHz): *δ* 8.13 (d, *J* = 5.3 Hz, 1H), 7.60 (d, *J* = 8.1 Hz, 2H), 7.51 (d, *J* = 8.0 Hz, 1H), 7.12 (dd, *J* = 8.3, 2.1 Hz, 1H), 7.00 (d, *J* = 2.1 Hz, 1H), 6.92 (d, *J* = 8.4 Hz, 1H), 6.83 (dd, *J* = 5.3, 1.5 Hz, 1H), 6.50 (d, *J* = 0.7 Hz, 1H), 5.07 (t, *J* = 5.8 Hz, 1H), 4.66 (d, *J* = 5.9 Hz, 2H), 3.91 (d, *J* = 7.8 Hz, 6H); ^13^C NMR (CDCl_3,_ 100 MHz) *δ* 154.9, 146.0, 145.8, 145.3, 144.7, 139.7, 127.8, 125.7, 125.4, 123.6, 121.7, 121.6, 118.9, 115.5, 108.2, 107.4, 106.0, 100.5, 52.1, 52.0, 41.9; HRMS *m*/*z* [M + H]^+^ calculated for C_21_H_19_F_3_N_2_O_2_: 389.1472; Found: 389.1486.

4-(3,4-Dimethoxyphenyl)-*N*-(3-(trifluoromethyl)benzyl)pyridin-2-amine (**6f**)

Yield: 22%, ^1^H NMR (CDCl_3_, 400 MHz): *δ* 8.14 (d, *J* = 5.3 Hz, 1H), 7.66 (s, 1H), 7.59 (d, *J* = 7.6 Hz, 1H), 7.54 (d, *J* = 7.8 Hz, 1H), 7.45 (t, *J* = 7.7 Hz, 1H), 7.13 (dd, *J* = 8.3, 2.1 Hz, 1H), 7.03 (d, *J* = 2.1 Hz, 1H), 6.92 (d, *J* = 8.4 Hz, 1H), 6.83 (dd, *J* = 5.3, 1.5 Hz, 1H), 6.53 (d, *J* = 0.7 Hz, 1H), 5.04 (t, *J* = 5.8 Hz, 1H), 4.66 (d, *J* = 5.9 Hz, 2H), 3.91 (d, *J* = 2.6 Hz, 6H); ^13^C NMR (CDCl_3,_ 100 MHz) *δ* 158.9, 149.9, 149.7, 149.2, 148.6, 140.6, 131.7, 131.1, 130.8, 129.1, 125.5, 124.2, 124.1, 124.1, 119.4, 112.2, 111.3, 109.9, 104.5, 56.0, 55.9, 45.9; HRMS *m*/*z* [M + H]^+^ calculated for C_21_H_19_F_3_N_2_O_2_: 389.1472; Found: 389.1483.

4-(3,4-Dimethoxyphenyl)-*N*-(2-(trifluoromethyl)benzyl)pyridin-2-amine (**6g**)

Yield: 20%, ^1^H NMR (CDCl_3_, 400 MHz): *δ* 8.14 (d, *J* = 5.8 Hz, 1H), 7.66–7.69 (m, 2H), 7.50 (t, *J* = 7.6 Hz, 1H), 7.50 (d, *J* = 7.6 Hz, 1H), 7.12 (dd, *J* = 8.3, 2.1 Hz, 1H), 7.03 (d, *J* = 2.1 Hz, 1H), 6.91 (d, *J* = 8.4 Hz, 1H), 6.83 (dd, *J* = 5.3, 1.5 Hz, 1H), 6.47 (d, *J* = 0.8 Hz, 1H), 5.08 (t, *J* = 6.1 Hz, 1H), 4.80 (d, *J* = 6.2 Hz, 2H), 3.91 (d, *J* = 2.5 Hz, 6H); ^13^C NMR (CDCl_3_, 100 MHz) *δ* 155.0, 145.9, 145.8, 145.3, 144.7, 134.2, 128.3, 127.7, 125.1, 124.1, 123.2, 122.1, 122.0, 115.4, 108.0, 107.4, 105.9, 100.0, 52.0, 51.9, 38.7; HRMS *m*/*z* [M + H]^+^ calculated for C_21_H_19_F_3_N_2_O_2_: 389.1472; Found: 389.1484.

### 4.3. Synthesis of (E)-1-(3,4-Dimethoxyphenyl)-3-(dimethylamino)prop-2-en-1-one (**9**)

3,4-Dimethoxyacetophenone **1** (1.11 mmol) was dissolved in DMF-DMA (10.0 mmol) and stirred under reflux for 1 h under a nitrogen stream. Then, the reaction mixture was extracted with DCM, water, the organic layer was washed with water, dried over anhydrous MgSO_4_, concentrated under reduced pressure, and purified by silica gel column chromatography (EA:Hex = 3:1) Yield 87%. ^1^H NMR (CDCl_3_, 400 MHz): *δ* 7.78 (d, *J* = 12.0 Hz, 1H), 7.55 (d, *J* = 1.6 Hz, 1H), 7.50 (dd, *J* = 8.4, 2.0 Hz, 1H) 6.85 (d, *J* = 8.4 Hz, 1H), 5.71 (d, *J* = 12.0 Hz, 1H), 3.94 (s, 3H), 3.92 (s, 3H), 3.02 (s, 6H).

### 4.4. General Procedure for the Synthesis of Pyrimidine Analogues (**10a**–**f**) of CGA

Benzylguanidine hydrochloride derivatives **8a**–**f** (0.46 mmol) and (*E*)-1-(3,4-dimethoxyphenyl)-3-(dimethylamino) prop-2-en-1-one (1.40 mmol) **9** was dissolved in EtOH (1.0 mL); then, potassium carbonate (1.40 mmol) was added, and stirred for 24 h under reflux. After completion of the reaction, aqueous NH_4_OAc was added, extracted with ethyl acetate, the organic layer was washed with water, dried over anhydrous MgSO_4_, concentrated under reduced pressure, and purified by silica gel column chromatography.

*N*-Benzyl-4-(3,4-dimethoxyphenyl)pyrimidin-2-amine (**10a**)

Yield: 40%, ^1^H NMR (CDCl_3_, 400 MHz): *δ* 8.32 (d, *J* = 5.3 Hz, 1H), 7.65 (d, *J* = 2.0 Hz, 1H), 7.58 (dd, *J* = 8.4, 2.0 Hz, 1H), 7.25–7.42 (m, 5H), 6.97 (d, *J* = 5.3 Hz, 1H), 6.93 (d, *J* = 8.4 Hz, 1H), 5.50 (t, *J* = 5.3 Hz, 1H), 4.73 (d, *J* = 5.92 Hz, 2H), 3.94 (d, *J* = 1.7 Hz, 6H); ^13^C NMR (CDCl_3,_ 100 MHz): *δ* 164.3, 162.5, 158.3, 151.3, 149.1, 139.6, 130.1, 128.6, 127.5, 127.2, 120.1, 110.8, 109.8, 106.3, 56.0, 55.9, 45.7; HRMS *m*/*z* [M + H]^+^ calculated for C_19_H_19_N_3_O_2_: 322.1550; Found: 322.1561.

*N*-(4-Chlorobenzyl)-4-(3,4-dimethoxyphenyl)pyrimidin-2-amine (**10b**)

Yield: 13%, ^1^H NMR (CDCl_3_, 500 MHz): *δ* 8.31 (d, *J* = 5.3 Hz, 1H), 7.61 (d, *J* = 2.0 Hz, 1H), 7.57 (dd, *J* = 8.4, 2.0 Hz, 1H), 7.26–7.35 (m, 4H), 6.98 (d, *J* = 5.3 Hz, 1H), 6.93 (d, *J* = 8.4 Hz, 1H), 5.52 (t, *J* = 5.5 Hz, 1H), 4.70 (d, *J* = 6.0 Hz, 2H), 3.94 (d, *J* = 4.8 Hz, 6H); ^13^C NMR (CDCl_3,_ 100 MHz) *δ* 164.4, 162.4, 158.3, 151.3, 149.2, 138.2, 132.8, 130.0, 128.8, 128.6, 120.1, 110.9, 109.7, 106.5, 56.0, 55.9, 44.9; HRMS *m*/*z* [M + H]^+^ calculated for C_19_H_18_ClN_3_O_2_: 356.1161; Found: 356.1171.

*N*-(3-Chlorobenzyl)-4-(3,4-dimethoxyphenyl)pyrimidin-2-amine (**10c**)

Yield: 41%, ^1^H NMR (CDCl_3_, 500 MHz): *δ* 8.30 (d, *J* = 5.3 Hz, 1H), 7.62 (d, *J* = 2.0 Hz, 1H), 7.56 (dd, *J* = 8.4, 2.0 Hz, 1H), 7.40 (s, 1H), 7.22–7.29 (m, 3H), 6.98 (d, *J* = 5.3 Hz, 1H), 6.93 (d,*J* = 8.4 Hz, 1H), 5.69 (s, 1H), 4.71 (d, *J* = 6.1 Hz, 2H), 3.94 (d, *J* = 3.4 Hz, 6H); ^13^C NMR (CDCl_3,_ 100 MHz) *δ* 164.3, 162.4, 158.3, 151.3, 149.1, 142.0, 134.3, 130.0, 129.8, 127.5, 127.2, 125.4, 120.1, 110.9, 109.7, 106.4, 56.0, 55.9, 45.0; HRMS *m*/*z* [M + H]^+^ calculated for C_19_H_18_ClN_3_O_2_: 356.1161; Found: 356.1171.

*N*-(2-Chlorobenzyl)-4-(3,4-dimethoxyphenyl)pyrimidin-2-amine (**10d**)

Yield: 44%, ^1^H NMR (CDCl_3_, 400 MHz): *δ* 8.31 (d, *J* = 5.3 Hz, 1H), 7.65 (d, *J* = 2.1 Hz, 1H), 7.57 (dd, *J* = 8.4, 2.0 Hz, 1H), 7.48–7.53 (m, 2H), 7.34–7.40 (m, 2H), 7.19–7.21 (m, 2H), 6.98 (d, *J* = 5.3 Hz, 1H), 6.92 (d, *J* = 8.4 Hz, 1H), 5.64 (t, *J* = 6.0 Hz, 1H), 4.82 (d, *J* = 6.3 Hz, 2H), 3.93 (d, *J* = 3.3 Hz, 6H); ^13^C NMR (CDCl_3_, 100 MHz) *δ* 164.3, 162.4, 158.4, 151.2, 149.1, 137.0, 133.4, 130.0, 129.4, 129.2, 128.4, 126.8, 120.1, 110.8, 109.7, 106.3, 56.0, 55.9, 43.4; HRMS *m*/*z* [M + H]^+^ calculated for C_19_H_18_ClN_3_O_2_: 356.1161; Found: 356.1170.

4-(3,4-Dimethoxyphenyl)-*N*-(4-(trifluoromethyl)benzyl)pyrimidin-2-amine (**10e**)

Yield: 07%, ^1^H NMR (CDCl_3_, 500 MHz): *δ* 8.32 (d, *J* = 5.3 Hz, 1H), 7.51–7.59 (m, 6H), 7.00 (d, *J* = 5.3 Hz, 1H), 6.93 (d, *J* = 8.4 Hz, 1H), 5.61 (t, *J* = 6.4 Hz, 1H), 4.79 (d, *J* = 6.1 Hz, 2H), 3.94 (s, 3H), 3.90 (s, 3H); ^13^C NMR (CDCl_3_, 100 MHz) *δ* 164.5, 162.4, 158.4, 151.4, 149.2, 143.9, 129.9, 127.5, 125.5, 125.5, 125.4, 120.1, 110.9, 109.7, 106.7, 56.0, 55.9, 45.1; HRMS *m*/*z* [M + H]^+^ calculated for C_20_H_18_F_3_N_3_O_2_: 390.1424; Found: 390.1438.

4-(3,4-Dimethoxyphenyl)-*N*-(3-(trifluoromethyl)benzyl)pyrimidin-2-amine (**10f**)

Yield: 27%, ^1^H NMR (CDCl_3_, 500 MHz): *δ* 8.32 (d, *J* = 5.3 Hz, 1H), 7.59–7.67 (m, 3H), 7.56 (dd, *J* = 8.4, 2.0 Hz, 1H), 7.52 (d, *J* = 7.8 Hz, 1H), 7.44 (t, *J* = 7.8 Hz, 1H), 7.99 (d, *J* = 5.3 Hz, 1H), 6.93 (d, *J* = 8.4 Hz, 1H), 5.62 (t, *J* = 5.6 Hz, 1H), 4.79 (d, *J* = 6.1 Hz, 2H), 3.93 (d, *J* = 12.2 Hz, 3H), 3.91 (s, 3H); ^13^C NMR (CDCl_3,_ 100 MHz) *δ* 164.4, 162.4, 158.3, 151.3, 149.1, 140.9, 130.9, 130.7, 130.6, 129.0, 124.1, 124.1, 123.9, 123.9, 122.8, 120.1, 110.8, 109.6, 106.5 56.0, 55.8, 45.1; HRMS *m*/*z* [M + H]^+^ calculated for C_20_H_18_F_3_N_3_O_2_: 390.1424; Found: 390.1436.

### 4.5. Synthesis of O-Acylated Derivatives of Acrylic Acid (**12a**–**12d**)

To synthesize acrylic acid derivatives **12a**–**d**, the starting material caffeic acid **11** (11.10 mmol) was dissolved in pyridine (10.0 mL) under nitrogen atm, DMAP (0.110 mmol) was added, and it was stirred at 0 °C for 10 min. Then, alkyl acid chlorides (554.14 mmol) were added dropwise and stirred at room temperature for 24 h. Once the reaction was complete, the organic layer was washed three times with ethyl acetate and 1N HCl; then, it was dried with MgSO_4_, filtered and concentrated. The pure product was obtained using silica gel column chromatography (EA:Hex = 1:1).

### 4.6. Synthesis of Cyclohexyl Ester Analogues **13a**–**d** of CGA

To a solution of **12a**–**d** (1.0 equiv.) in toluene (1.0 mL), SOCl_2_ (5.0 equiv.) was added dropwise and stirred at rt for 3 h. Once the starting materials were consumed, the excess SOCl_2_ and solvent were removed. Then, the residue was dissolved in DCM (2.0 mL) and DMAP (0.2 equiv.) was added; and stirring was continued for 30 more minutes. Next, cyclohexanol (1.0 equiv.) was added to the reaction mixture and stirred for additional 12 at rt. After the reaction was completed, the solvent was removed under reduced pressure, and the obtained crude product was purified by using silica gel column chromatography to afford **13a**–**d**.

(*E*)-4-(3-(Cyclohexyloxy)-3-oxoprop-1-en-1-yl)-1,2-phenylene dibutyrate (**13a**)

Yield: 34%, ^1^H NMR (CDCl_3_, 400 MHz) *δ* 7.60 (d, *J* = 16.0 Hz, 1H), 7.39 (dd, *J* = 8.4, 2.0 Hz, 1H), 7.35 (d, *J* = 2.0 Hz, 1H), 7.21 (d, *J* = 8.4 Hz, 1H), 6.38 (d, *J* = 16.0 Hz, 1H), 4.88 (m, 1H), 2.53 (td, *J* = 7.2, 2.4 Hz, 4H), 1.92 (m, 2H), 1.83 (m, 6H), 1.83–1.23 (m, 6H), 1.05 (td, *J* = 7.2, 2.4 Hz, 6H); ^13^C NMR (CDCl_3,_ 100 MHz) *δ* 170.8, 170.8, 166.1, 143.5, 142.5, 142.4, 133.3, 126.2, 123.9, 122.7, 120.0, 72.9, 35.9, 35.9, 31.7, 25.4, 32.8, 18.4, 13.7, 13.7; HRMS *m*/*z* [M + Na]^+^ calculated for C_23_H_30_O_6_: 425.1934, found: 425.1933.

(*E*)-4-(3-(Cyclohexyloxy)-3-oxoprop-1-en-1-yl)-1,2-phenylene dihexanoate (**13b**)

Yield: 58%, ^1^H NMR (CDCl_3_, 400 MHz) *δ* 7.59 (d, *J* = 16.0 Hz, 1H), 7.37 (dd, *J* = 8.4, 2.0 Hz, 1H), 7.34 (d, *J* = 2.0 Hz, 1H), 7.19 (d, *J* = 8.4 Hz, 1H), 6.36 (d, *J* = 16.0 Hz, 1H) 4.88 (1H, m), 2.52 (td, *J* = 7.6, 2.8 Hz, 1H), 1.90–0.89 (m, 32H); ^13^C NMR (CDCl_3,_ 100 MHz) *δ* 170.9, 170.8, 166.1, 143.5, 142.6, 142.4, 133.3, 126.2, 123.9, 122.7, 120.0, 72.9, 34.1, 34.0, 31.7, 31.3, 25.5, 24.6, 23.8, 22.4, 13.9; HRMS *m*/*z* [M + Na]^+^ calculated for C_27_H_38_O_6_: 481.2560, Found: 481.2559.

(*E*)-4-(3-(Cyclohexyloxy)-3-oxoprop-1-en-1-yl)-1,2-phenylene bis(2,2-dimethylpropanoate) (**13c**)

Yield: 12%, ^1^H NMR (CDCl_3_, 400 MHz) *δ* 7.62 (d, *J* = 16.0 Hz, 1H), 7.37 (dd, *J* = 8.4, 2.4 Hz, 1H), 7.29 (d, *J* = 2.4 Hz, 1H), 7.15 (d, *J* = 8.4 Hz, 1H), 6.37 (d, *J* = 16.0 Hz, 1H) 4.88 (1H, m), 1.89–1.39 (m, 4H), 1.36–1.29 (m, 26H); ^13^C NMR (CDCl_3_, 100 MHz) *δ* 175.8, 175.7, 166.1, 144.1, 143.0, 142.5, 133.2, 126.1, 123.9, 122.7, 119.9, 72.9, 39.3, 39.3, 31.8, 27.3, 27.3, 25.5, 23.9; HRMS *m*/*z* [M + Na]^+^ calculated for C_25_H_34_O_6_: 453.2247, found: 453.2246.

(*E*)-4-(3-(Cyclohexyloxy)-3-oxoprop-1-en-1-yl)-1,2-phenylene dicyclohexanecarboxylate (**13d**)

Yield: 31%, ^1^H NMR (CDCl_3_, 400 MHz) *δ* 7.58 (d, *J* = 16 Hz, 1H), 7.37–7.26 (m, 2H), 7.16 (d, *J* = 8.4 Hz, 1H), 7.15 (d, *J* = 8.4 Hz, 1H), 6.35 (d, *J* = 16.0 Hz, 1H) 4.86 (1H, m), 2.52 (m, 2H), 2.04–1.30 (m, 30H); ^13^C NMR (CDCl_3,_ 100 MHz) *δ* 173.1, 173.1, 166.1, 143.7, 142.7, 142.5, 133.2, 126.1, 123.9, 122.7, 119.9, 77.3, 72.9, 43.1, 43.0, 31.7, 29.0, 25.7, 25.4, 25.3, 23.8; HRMS *m*/*z* [M + Na]^+^ calculated for C_29_H_38_O_6_: 505.2560, Found: 505.2557.

### 4.7. Synthesis of (E)-Cyclohexyl 3-(3,4-dihydroxyphenyl)acrylate (**14**)

To the caffeic acid (**11**) (0.10 g, 0.55 mmol) in cyclohexanol (20 mL), sulfuric acid (0.2 mL, 3.7 mmol) was added dropwise and stirred at reflux for 1 h. Reaction progress was monitored by using TLC. After that, it was diluted with ethyl acetate and washed three times with NaHCO_3_ solution and three times with aqueous NaCl solution. The organic layers were collected and dried by using MgSO_4_, and solvent was evaporated under reduced pressure. The obtained crude reaction mixture was purified by silica gel column chromatography (EA:Hx = 1:3) to give compound **14** as a brown solid.

Yield: 62%, ^1^H NMR (CDCl_3_, 400 MHz) *δ* 7.55 (d, 1H, *J* = 16.0 Hz), 7.08 (d, 1H, *J* = 1.6 Hz), 7.01 (dd, *J* = 8.4, 2.0 Hz), 6.86 (d, *J* = 8.4 Hz), 6.25 (d, *J* = 16.0 Hz), 5.96 (s, 1H), 5.78 (s, 1H), 4.88 (m, 1H), 2.0–1.7 (m, 10H).

### 4.8. Synthesis of (E)-Cyclohexyl 3-(3,4-diisobutoxyphenyl)acrylate (**15**)

To the (*E*)-cyclohexyl 3-(3,4-dihydroxyphenyl) acrylate **14** (50 mg, 0.19 mmol) in DMF (1 mL), K_2_CO_3_ (130 mg, 0.95 mmol) was added and stirred at 70 °C for 10 min; after that, 1-bromo-2-methylpropane (0.05 mL, 0.47 mmol) was added and stirred for 24 h. The organic layer was extracted three times with CH_2_Cl_2_, washed three times with water and aqueous NaCl solution, and then dried with MgSO_4_ and concentrated by filtration. Then, it was purified by column chromatography (MC:Hx = 1:1) to obtain compound **15** as a white solid.

Yield: 85%, ^1^H NMR (CDCl_3_, 400 MHz) *δ* 7.59 (d, *J* = 16.0 Hz, 1H), 7.05 (m, 2H), 6.83 (d, *J* = 8.8 Hz, 1H), 6.28 (d, 16.0 Hz, 1H), 4.87 (m, 1H), 3.78 (d, 4.0 Hz, 2H), 3.77 (d, 4.0 Hz, 2H), 2.14 (m, 2H), 1.94–1.92 (m, 10H), 1.05 (d, 3.2 Hz, 6H), 1.04 (d, 3.2 Hz, 6H); ^13^C NMR (CDCl_3,_ 100 MHz) *δ* 166.8, 151.5, 149.4, 144.4, 127.4, 122.6, 116.2, 113.0, 112.1, 75.6, 75.3, 72.5, 31.8, 28.4, 28.4, 25.5, 23.9, 19.3, 19.2; HRMS *m*/*z* [M + Na]^+^ calculated for C_23_H_34_O_4_: 397.2349, found: 397.2341.

### 4.9. Synthesis of (E)-Cyclohexyl 3-(3,4-dimethoxyphenyl)acrylate (**17**)

To the solution of 3,4-dimethoxycinnamic acid **16** (0.48 mmol) in toluene (1 mL), thionyl chloride (0.17 mL, 2.4 mmol) was added under nitrogen atm, and the mixture was stirred under reflux for 1.5 h. Then, after removing thionyl chloride, it was dissolved in anhydrous CH_2_Cl_2_ under nitrogen atm, and then DMAP (8 mg, 0.049 mmol) was added, and stirring continued for 10 min. Then, cyclohexanol (0.05 mL, 0.48 mmol) was added dropwise to the reaction mixture and stirred at room temperature for 24 h. The reaction mixture was poured into 10 mL of water and extracted with DCM. Dried with MgSO_4_, solvent was evaporated to obtain crude reaction mixture. This was purified by silica gel column chromatography (EA:Hx = 1:6) to obtain compound **17**.

Yield: 71%, ^1^H NMR (CDCl_3_, 400 MHz) *δ* 7.63 (d, *J* = 16.0 Hz, 1H), 7.10 (dd, *J* = 8.0, 2.0 Hz, 1H), 7.05 (d, *J* = 2.0 Hz, 1H), 6.86 (d, *J* = 8.4 Hz, 1H), 6.31 (d, *J* = 16.0 Hz, 1H), 4.88 (m, 1H), 3.90 (s, 6H), 1.94–1.24 (m, 10H); ^13^C NMR (CDCl_3,_ 100 MHz) *δ* 166.7, 151.9, 149.3, 144.3, 127.6, 122.6, 116.7, 111.1, 109.6, 72.7, 56.0, 55.9, 31.9, 25.5, 23.9; HRMS *m*/*z* [M + Na]^+^ calculated for C_17_H_22_O_4_: 313.1410, found: 313.1401.

### 4.10. Biological Assay 

#### 4.10.1. Melanin Measurement

Mouse melanoma B16 cells were cultured in a 96-well culture plate (2.5 × 10^3^ cells/well) [63]. Culture media were washed from each culture plates after 24 h and treated with freshly prepared 10% FBS-DMEM medium, various concentrations (such as 0.5, 1.0, 3.0, 10.0, 30.0, and 100.0 µM) of compound samples, and stimulants (α-MSH, 10.0 nM). Then, it was incubated at 37 °C for 72 h, and the amount of melanin released into the medium was determined by measuring the absorbance at wavelength of 405 nm using spectrophotometer (Molecular Device, San Jose, CA, USA). The absorbance of stimulated cells using α-MSH was used as a control, and the inhibition level was calculated by subtracting the absorbance values of cells that were not stimulated. The absorbance values were compared with standard curves obtained with synthetic melanin. The synthetic melanin (Sigma-Aldrich, St. Louis, MO, USA) was dissolved in 0.85 M KOH-10% DMSO solution; then, it was serially diluted with 10% FBS-DMEM medium and then aliquoted into a 96-well culture plate. Then, the absorbance of melanin was measured by a spectrophotometer at 405 nm, and a standard curve was prepared. Using this value, the amount of melanin released out of the cell was directly calculated. Results obtained by three or more sets of experiments were expressed as mean ± standard error.

#### 4.10.2. MTT Assay 

Melanoma B16 cells were seeded into a 96-well culture plate at a seeding density of 2.5 × 10^3^ cells/well and incubated for 24 h at 37 °C supplied with 5% CO_2_ [68]. The culture medium of each well was replaced with a fresh 10% FBS-DMEM medium and treated with serially diluted samples and α-MSH. After 72 h of incubation, cells were incubated with fresh 10% FBS-DMEM medium. After 2 h of stabilization, 100 μL of 1 mg/mL MTT solution was added per well to lyse the cells; then, the absorbance at 590 nm was measured using a spectrophotometer. The experiments were carried out three or more times, and the results were expressed as mean ± standard error.

### 4.11. Stability of CGA Acyl Analogues in Methanol Solution

The stability of the synthesized acyl analogues was measured in methanol, since the acetyl analogue of CGA was highly unstable in methanol solution. First, 10 mg of the sample was dissolved in 2 mL of methanol and stored at 25 °C. Then, purity was analyzed using HPLC (Shimadzu LC solution, Kyoto, Japan) for 28 days. The samples in methanol solution were diluted 10 times in the HPLC mobile phase ((A) 0.5% formic acid in H_2_O (B) 0.5% formic acid in acetonitrile (ACN), A/B = 20/80) and injected in HPLC column (inertsil ODS-3V C18 column (250 × 4.00 mm i.d; 5 μm)). Finally, the amount of acyl compound remaining in the solution was measured by calculating the area under the curve of the HPLC chromatogram, and the results were expressed in percentage units.

## 5. Conclusions

We have developed the synthesis method of CGA analogues comprising pyridine, pyrimidine motifs, as well as cyclohexyl ester analogues in moderate to good yields. Among the nineteen synthesized compounds, fifteen compounds displayed potent inhibition activity against melanin production in B16 melanoma cells upon α-MSH stimulation. Results showed that pyridine containing **6b**, **6c**, **6f** analogues, and diacyl CGA cyclohexyl ester analogues **13a**–**d** have greatly superior inhibition potency than the positive control, kojic acid. In addition, analogues **13c** and **13d** showed high stability profile in methanol solution compared to analogue **18** in 28 days of stability monitoring.

## Figures and Tables

**Table 1:** Inhibitory activity of pyridine (**6a**–**g**), pyrimidine (**10a**–**f**), and cyclohexyl ester (**13a**–**13d**, **15** and **17**) analogues of CGA against α-MSH-induced melanogenesis in B16 melanoma cells.

Compounds	R	IC_50_ (μM) ^a,c^
Kojic acid ^b^	-	54.0 ± 1.5
Arbutin ^b^	-	380.0 ± 9.5
**6a**	H	17.0 ± 4.0
**6b**	4-Cl	4.5 ± 0.7
**6c**	3-Cl	5.0 ± 1.4
**6d**	2-Cl	8.0 ± 1.0
**6e**	4-CF_3_	8.5 ± 0.7
**6f**	3-CF_3_	2.5 ± 0.7
**6g**	2-CF_3_	7.5 ± 0.7
**10a**	H	>100
**10b**	4-Cl	54.0 ± 6.0
**10c**	3-Cl	32.0 ± 2.0
**10d**	2-Cl	40.0 ± 8.0
**10e**	4-CF_3_	>100
**10f**	3-CF_3_	20.0 ± 1.0
**13a**	*n*-Pr	1.0 ± 0.1
**13b**	*n*-Pent	1.3 ± 0.4
**13c**	*t*-But	1.9 ± 0.1
**13d**	Cy	2.3 ± 0.2
**15**	-	>100
**17**	-	>100

^a^ Data are displayed as mean ± SD (n = 2), ^b^ Kojic acid and arbutin were used as the positive control, ^c^ Cytotoxicity at >30 μM for all the tested analogues (except **6f**).

**Table 2:** Stability of CGA acyl analogues in methanol over the time (28 days) ^a^.

	Acyl Analogues				
Days	18	13a	13b	13c	13d
0	99.5029	99.3294	99.8006	99.5492	98.9750
1	71.1918	91.3520	93.7356	99.3034	96.9973
2	55.5162	85.3800	89.9637	99.1518	95.8267
3	43.5699	80.3685	86.8066	99.0038	94.6557
4	33.7154	75.4921	83.9188	98.8061	93.5713
5	27.3752	72.147	82.1086	98.5395	93.0573
7	16.5047	64.7751	77.5962	97.9332	91.6229
9	10.5572	59.4842	74.0389	97.4173	90.6724
11	6.9200	55.1819	70.6843	96.8033	89.8768
21	1.4408	43.3505	58.4127	95.0243	87.5571
28	0.6625	39.0229	52.5500	94.0833	86.5501

^a^ 10 mg sample was dissolved in methanol and left at 25 °C. Purity/stability of each day was determined by HPLC analysis.

## Data Availability

Data are contained within the article or associated supplementary files.

## Supplementary Materials

The following are available online at https://www.mdpi.com/article/10.3390/ph14111176/s1, ^1^H and ^13^C NMR spectra for all the synthesized compounds, IC_50_ values.

## References

[1] Pillaiyar T., Namasivayam V., Manickam M., Jung S.-H. (2018). Inhibitors of Melanogenesis: An Updated Review. J. Med. Chem..

[2] Pillaiyar T., Manickam M., Namasivayam V. (2017). Skin whitening agents: Medicinal chemistry perspective of tyrosinase inhibitors. J. Enzym. Inhib. Med. Chem..

[3] Imokawa G., Ishida K. (2014). Inhibitors of Intracellular Signaling Pathways that Lead to Stimulated Epidermal Pigmentation: Perspective of Anti-Pigmenting Agents. Int. J. Mol. Sci..

[4] Videira I.F.S., Lima Moura D.F., Vasconcelos Magina S.B.L.M. (2013). Mechanisms regulating melanogenesis. An. Bras. Dermatol..

[5] Gillbro J.M., Olsson M.J. (2011). The melanogenesis and mechanisms of skin-lightening agents–existing and new approaches. Int. J. Cosmet. Sci..

[6] Obaid R.J., Mughal E.U., Naeem N., Sadiq A., Alsantali R.I., Jassas R.S., Moussa Z., Ahmed S.A. (2021). Natural and synthetic flavonoid derivatives as new potential tyrosinase inhibitors: A systematic review. RSC Adv..

[7] Li J., Feng L., Liu L., Wang F., Ouyang L., Zhang L., Hu X., Wang G. (2021). Recent advances in the design and discovery of synthetic tyrosinase inhibitors. Eur. J. Med. Chem..

[8] Yuan Y., Jin W., Nazir Y., Fercher C., Blaskovich M.A.T., Cooper M.A., Barnard R.T., Ziora Z.M. (2020). Tyrosinase inhibitors as potential antibacterial agents. Eur. J. Med. Chem..

[9] Lai X., Wichers H.J., Soler-Lopez M., Dijkstra B.W. (2018). Structure and Function of Human Tyrosinase and Tyrosinase-Related Proteins. Chem. Eur. J..

[10] Hasegawa K., Fujiwara R., Sato K., Shin J., Kim S.J., Kim M., Kang H.Y. (2015). Possible involvement of keratinocyte growth factor in the persistence of hyperpigmentation in both human facial solar lentigines and melasma. Ann. Dermatol..

[11] Savoye I., Olsen C.M., Whiteman D.C., Bijon A., Wald L., Dartois L., Clavel-Chapelon F., Boutron-Ruault M.C., Kvaskoff M. (2018). Patterns of Ultraviolet Radiation Exposure and Skin Cancer Risk: The E3N-SunExp Study. J. Epidemiol..

[12] Ortonne J.P., Bissett D.L. (2008). Latest insights into skin hyperpigmentation. J. Investig. Dermatol. Symp. Proc..

[13] Bastiaens M., Hoefnagel J., Westendorp R., Vermeer B.J., Bavinck J.N.B. (2004). Solar lentigines are strongly related to sun exposure in contrast to ephelides. Pigment Cell Res..

[14] Niu C., Aisa H.A. (2017). Upregulation of Melanogenesis and Tyrosinase Activity: Potential Agents for Vitiligo. Molecules.

[15] De Gruijl F.R. (1999). Skin cancer and solar UV radiation. Eur. J. Cancer.

[16] Neagu E., Radu G.L., Albu C., Paun G. (2018). Antioxidant activity, acetylcholinesterase and tyrosinase inhibitory potential of Pulmonaria officinalis and Centarium umbellatum extracts. Saudi J. Biol. Sci..

[17] Jiang H., Newman M., Lardelli M. (2018). The zebrafish orthologue of familial Alzheimer’s disease gene PRESENILIN 2 is required for normal adult melanotic skin pigmentation. PLoS ONE.

[18] Lavezzo M.M., Sakata V.M., Morita C., Rodriguez E.E.C., Abdallah S.F., da Silva F.T.G., Hirata C.E., Yamamoto J.H. (2016). Vogt-Koyanagi-Harada disease: Review of a rare autoimmune disease targeting antigens of melanocytes. Orphanet J. Rare Dis..

[19] https://cosmetics.specialchem.com/news/industry-news/skin-lightening-products-market-to-reach-usd23-bn-by-2020-global-industry-analysts.

[20] Iraji A., Panahi Z., Edraki N., Khoshneviszadeh M., Khoshneviszadeh M. (2021). Design, synthesis, in vitro and in silico studies of novel Schiff base derivatives of 2-hydroxy-4-methoxybenzamide as tyrosinase inhibitors. Drug Dev. Res..

[21] Hosseinpoor H., Moghadam Farid S., Iraji A., Askari S., Edraki N., Hosseini S., Jamshidzadeh A., Larijani B., Attarroshan M., Pirhadi S. (2021). Anti-melanogenesis and anti-tyrosinase properties of aryl-substituted acetamides of phenoxy methyl triazole conjugated with thiosemicarbazide: Design, synthesis and biological evaluations. Bioorg. Chem..

[22] Gao D., Kim J.H., Kim C.T., Jeong W.S., Kim H.M., Sim J., Kang J.S. (2021). Evaluation of Anti-Melanogenesis Activity of Enriched Pueraria lobata Stem Extracts and Characterization of Its Phytochemical Components Using HPLC–PDA–ESI–MS/MS. Int. J. Mol. Sci..

[23] Durai P., Ko Y.-J., Kim J.-C., Pan C.-H., Park K. (2021). Identification of Tyrosinase Inhibitors and Their Structure-Activity Relationships via Evolutionary Chemical Binding Similarity and Structure-Based Methods. Molecules.

[24] Song S., Mai Y., Shi H., Liao B., Wang F. (2020). Design, Synthesis, Biological Evaluation and Inhibition Mechanism of 3-/4-Alkoxy Phenylethylidenethiosemicarbazides as New, Potent and Safe Tyrosinase Inhibitors. Chem. Pharm. Bull..

[25] Hosseinpoor H., Iraji A., Edraki N., Pirhadi S., Attarroshan M., Khoshneviszadeh M., Khoshneviszadeh M. (2020). A Series of Benzylidenes Linked to Hydrazine-1-carbothioamide as Tyrosinase Inhibitors: Synthesis, Biological Evaluation and Structure–Activity Relationship. Chem. Biodivers..

[26] Dettori M.A., Fabbri D., Dessì A., Dallocchio R., Carta P., Honisch C., Ruzza P., Farina D., Migheli R., Serra P.A. (2020). Synthesis and Studies of the Inhibitory Effect of Hydroxylated Phenylpropanoids and Biphenols Derivatives on Tyrosinase and Laccase Enzymes. Molecules.

[27] Ullah S., Kang D., Lee S., Ikram M., Park C., Park Y., Yoon S., Chun P., Moon H.R. (2019). Synthesis of cinnamic amide derivatives and their anti-melanogenic effect in α-MSH-stimulated B16F10 melanoma cells. Eur. J. Med. Chem..

[28] Hałdys K., Latajka R. (2019). Thiosemicarbazones with tyrosinase inhibitory activity. Med. Chem. Comm..

[29] Arepalli S.K., Lee C., Jung J.K., Kim Y., Lee K., Lee H. (2019). Synthesis of N-arylindazole-3-carboxamide and N-benzoylindazole derivatives and their evaluation against α-MSH-stimulated melanogenesis. Bioorg. Med. Chem. Lett..

[30] He M., Fan M., Liu W., Li Y., Wang G. (2021). Design, synthesis, molecular modeling, and biological evaluation of novel kojic acid derivatives containing bioactive heterocycle moiety as inhibitors of tyrosinase and antibrowning agents. Food Chem..

[31] Cardoso R., Valente R., Souza da Costa C.H., da S. (2021). Gonçalves Vianez, J.L.; Santana da Costa, K.; de Molfetta, F.A.; Nahum Alves, C. Analysis of Kojic Acid Derivatives as Competitive Inhibitors of Tyrosinase: A Molecular Modeling Approach. Molecules.

[32] Ashooriha M., Khoshneviszadeh M., Khoshneviszadeh M., Rafiei A., Kardan M., Yazdian-Robati R., Emami S. (2020). Kojic acid–natural product conjugates as mushroom tyrosinase inhibitors. Eur. J. Med. Chem..

[33] Hałdys K., Goldeman W., Anger-Góra N., Rossowska J., Latajka R. (2021). Monosubstituted acetophenone thiosemicarbazones as potent inhibitors of tyrosinase: Synthesis, inhibitory studies, and molecular docking. Pharmaceuticals.

[34] Ketata E., Elleuch H., Neifar A., Mihoubi W., Ayadi W., Marrakchi N., Rezgui F., Gargouri A. (2019). Anti-melanogenesis potential of a new series of Morita-Baylis-Hillman adducts in B16F10 melanoma cell line. Bioorg. Chem..

[35] Sepehri N., Iraji A., Yavari A., Asgari M.S., Zamani S., Hosseini S., Bahadorikhalili S., Pirhadi S., Larijani B., Khoshneviszadeh M. (2021). The natural-based optimization of kojic acid conjugated to different thio-quinazolinones as potential anti-melanogenesis agents with tyrosinase inhibitory activity. Bioorg. Med. Chem..

[36] Mahajan P.G., Dige N.C., Vanjare B.D., Raza H., Hassan M., Seo S.-Y., Kim C.-H., Lee K.H. (2019). Facile synthesis of new quinazolinone benzamides as potent tyrosinase inhibitors: Comparative spectroscopic and molecular docking studies. J. Mol. Struct..

[37] Tang K., Jiang Y., Zhang H., Huang W., Xie Y., Deng C., Xu H., Song X., Xu H. (2021). Design, synthesis of Cinnamyl-paeonol derivatives with 1, 3-Dioxypropyl as link arm and screening of tyrosinase inhibition activity in vitro. Bioorg. Chem..

[38] Gaikwad N., Nanduri S., Madhavi Y.V. (2019). Cinnamamide: An insight into the pharmacological advances and structure–activity relationships. Eur. J. Med. Chem..

[39] Lončar B., Perin N., Mioč M., Boček I., Grgić L., Kralj M., Tomić S., Stojković M.R., Hranjec M. (2021). Novel amino substituted tetracyclic imidazo[4,5-*b*]pyridine derivatives: Design, synthesis, antiproliferative activity and DNA/RNA binding study. Eur. J. Med. Chem..

[40] Lee S., Kwon N.H., Seo B., Lee J.Y., Cho H.Y., Kim K., Kim H.S., Jung K., Jeon Y.H., Kim S. (2021). Discovery of novel potent migrastatic Thiazolo[5,4-*b*]pyridines targeting Lysyl-tRNA synthetase (KRS) for treatment of Cancer metastasis. Eur. J. Med. Chem..

[41] Krajčovičová S., Jorda R., Vanda D., Soural M., Kryštof V. (2021). 1,4,6-Trisubstituted imidazo[4,5-*c*]pyridines as inhibitors of Bruton’s tyrosine kinase. Eur. J. Med. Chem..

[42] Sahu M., Siddiqui N. (2016). A review on biological importance of pyrimidines in the new era. J. Pharm. Pharm. Sci..

[43] Kaur R., Chaudhary S., Kumar K., Gupta M.K., Rawal R.K. (2017). Recent synthetic and medicinal perspectives of dihydropyrimidinones: A review. Eur. J. Med. Chem..

[44] Rani J., Kumar S., Saini M., Mundlia J., Verma P.K. (2016). Biological potential of pyrimidine derivatives in a new era. Res. Chem. Intermed..

[45] Gheibi N., Taherkhani N., Ahmadi A., Haghbeen K., Ilghari D. (2015). Characterization of inhibitory effects of the potential therapeutic inhibitors, benzoic acid and pyridine derivatives, on the monophenolase and diphenolase activities of tyrosinase. Iran. J. Med. Sci..

[46] Choi J., Park S.-J., Jee J.-G. (2015). Analogues of ethionamide, a drug used for multidrug-resistant tuberculosis, exhibit potent inhibition of tyrosinase. Eur. J. Med. Chem..

[47] Bellei B., Pitisci A., Migliano E., Cardinali G., Picardo M. (2014). Pyridinyl imidazole compounds interfere with melanosomes sorting through the inhibition of Cyclin G-associated Kinase, a regulator of cathepsins maturation. Cell. Signal..

[48] Hseu Y.-C., Chen X.-Z., Vudhya Gowrisankar Y., Yen H.-R., Chuang J.-Y., Yang H.-L. (2020). The Skin-Whitening Effects of Ectoine via the Suppression of α-MSH-Stimulated Melanogenesis and the Activation of Antioxidant Nrf2 Pathways in UVA-Irradiated Keratinocytes. Antioxidants.

[49] Mirmortazavi S.S., Farvandi M., Ghafouri H., Mohammadi A., Shourian M. (2019). Evaluation of novel pyrimidine derivatives as a new class of mushroom tyrosinase inhibitor. Drug Des. Devel. Ther..

[50] Chung Y.C., Kim M.-J., Kang E.Y., Kim Y.B., Kim B.S., Park S.-M., Hyun C.-G. (2019). Anti-Melanogenic Effects of Hydroxyectoine via MITF Inhibition by JNK, p38, and AKT Pathways in B16F10 Melanoma Cells. Nat. Prod. Commun..

[51] Lu H., Tian Z., Cui Y., Liu Z., Ma X. (2020). Chlorogenic acid: A comprehensive review of the dietary sources, processing effects, bioavailability, beneficial properties, mechanisms of action, and future directions. Compr. Rev. Food Sci. Food Saf..

[52] Kim H.H., Kim J.K., Kim J., Jung S.-H., Lee K. (2020). Characterization of Caffeoylquinic Acids from Lepisorus thunbergianus and Their Melanogenesis Inhibitory Activity. ACS Omega.

[53] Li H.-R., Habasi M., Xie L.-Z., Aisa H.A. (2014). Effect of Chlorogenic Acid on Melanogenesis of B16 Melanoma Cells. Molecules.

[54] Kumar R., Sharma A., Iqbal M.S., Srivastava J.K. (2020). Therapeutic promises of chlorogenic acid with special emphasis on its anti-obesity property. Curr. Mol. Pharmacol..

[55] Tajik N., Tajik M., Mack I., Enck P. (2017). The potential effects of chlorogenic acid, the main phenolic components in coffee, on health: A comprehensive review of the literature. Eur. J. Nutr..

[56] Plazas M., Andújar I., Vilanova S., Hurtado M., Gramazio P., Herraiz F.J., Prohens J. (2013). Breeding for Chlorogenic Acid Content in Eggplant: Interest and Prospects. Not. Bot. Horti Agrobot. Cluj-Napoca.

[57] Shi H., Dong L., Jiang J., Zhao J., Zhao G., Dang X., Lu X., Jia M. (2013). Chlorogenic acid reduces liver inflammation and fibrosis through inhibition of toll-like receptor 4 signaling pathway. Toxicology.

[58] Yun N., Kang J.-W., Lee S.-M. (2012). Protective effects of chlorogenic acid against ischemia/reperfusion injury in rat liver: Molecular evidence of its antioxidant and anti-inflammatory properties. J. Nutr. Biochem..

[59] Zhang X., Huang H., Yang T., Ye Y., Shan J., Yin Z., Luo L. (2010). Chlorogenic acid protects mice against lipopolysaccharide-induced acute lung injury. Injury.

[60] Anqi Z., Xin L., Shaomi Z., Chi L., Shu W., Qinxiu Z., Junning Z., Linjiang S. (2021). Chlorogenic acid induces apoptosis, inhibits metastasis and improves antitumor immunity in breast cancer via the NF-κB signaling pathway. Oncol. Rep..

[61] Luigi S., Alessia S., Michela I., Angela R., Annamaria S., Emilio C., Severina P., Michelina C., Silvio N. (2020). Chlorogenic acid activates ERK1/2 and inhibits proliferation of osteosarcoma cells. J. Cell. Physiol..

[62] Alessia S., Angela R., Spina A., Silvio N., Luigi S. (2021). Chlorogenic Acid Enhances Doxorubicin-Mediated Cytotoxic Effect in Osteosarcoma Cells. Int. J. Mol. Sci..

[63] Jo H., Choi M., Sim J., Viji M., Li S., Lee Y.H., Kim Y., Seo S.Y., Zhou Y., Lee K. (2017). Synthesis and biological evaluation of caffeic acid derivatives as potent inhibitors of α-MSH-stimulated melanogenesis. Bioorg. Med. Chem. Lett..

[64] Jo H., Zhou Y., Viji M., Choi M., Lim J.Y., Sim J., Rhee J., Kim Y., Seo S.Y., Kim W.J. (2017). Synthesis, biological evaluation, and metabolic stability of chlorogenic acid derivatives possessing thiazole as potent inhibitors of α-MSH-stimulated melanogenesis. Bioorg. Med. Chem. Lett..

[65] Sim J., Viji M., Rhee J., Jo H., Cho S.J., Park Y., Seo S.Y., Jung K.Y., Lee H., Jung J.K. (2019). γ-Functionalization of α,β-Unsaturated Nitriles under Mild Conditions: Versatile Synthesis of 4-Aryl-2-Bromopyridines. Adv. Synth. Catal..

[66] An T., Kang B., Kang S., Pac J., Youk J., Lin D., Lee Y. (2019). Guanidine cyclic diimides and their polymers. Chem. Commun..

[67] Lingel A., Sendzik M., Huang Y., Shultz M.D., Cantwell J., Dillon M.P., Fu X., Fuller J., Gabriel T., Gu J. (2017). Structure-Guided Design of EED Binders Allosterically Inhibiting the Epigenetic Polycomb Repressive Complex 2 (PRC2) Methyltransferase. J. Med. Chem..

[68] Thanigaimalai P., Lee K.C., Bang S.C., Lee J.H., Yun C.Y., Roh E., Hwang B.Y., Kim Y., Jung S.H. (2010). Inhibitory effect of novel tetrahydropyrimidine-2(1*H*)-thiones on melanogenesis. Bioorg. Med. Chem..

