# Supplementary material for: Inhibitory Effect of Chlorogenic Acid Analogues Comprising Pyridine and Pyrimidine on α-MSH-Stimulated Melanogenesis and Stability of Acyl Analogues in Methanol
Source: Pharmaceuticals (Basel). 2021 Nov 17;14(11):1176. doi: 10.3390/ph14111176 (PMC8622415; doi:10.3390/ph14111176)
Supplement: Supplementary file 1 [file pharmaceuticals-14-01176-s001.zip › pharmaceuticals-1425955-supplementary.pdf]

# Supporting Information

# Inhibitory effect of chlorogenic acid analogues comprising pyridine and pyrimidine on α-MSH-stimulated melanogenesis and stability of acyl analogues

in methanol Jaeuk Sim,[a,†] Lanka Srinu,[a,†] Jo Jeongwoong,[a] Chhabi Lal Chaudhary,[a] Manjunatha Vishwanath,[a] Chanhyun Jung,[a] Young Hee Lee,[a,b] Eun-yeong Kim,[c] Youngsoo Kim,[a] Soonsil Hyun,[a] Heesoon Lee,[a] Kiho Lee,[c] Seung-Yong Seo,[d] Mayavan Viji,[a,*] Jae-Kyung Jung,[a,*]

[a] *College of Pharmacy and Medicinal Research Center (MRC), Chungbuk National University, Cheongju 28160, Republic of Korea*

[b] *Samjin Central Research Institute, Samjin Pharma Co., LTD, Cheongju 28158, Republic of Korea*

[c] *College of Pharmacy, Korea University, Sejong 30019, Republic of Korea*

[d] *College of Pharmacy and Gachon Institute of Pharmaceutical Sciences, Gachon University, Incheon 21936, Republic of Korea*

[†] *both* the authors equally contributed

Corresponding author. Tel.: +82-43-261-2635; fax: +82-43-268-2732. E-mail: orgjkjung@chungbuk.ac.kr (J.-K. Jung); Email: cheviji@gmail.com (M. Viji)

**Contents** **Page**

1. **NMR Spectra** **2**

2. **$IC_{50}$ Values** **21**

# 1. NMR Spectra

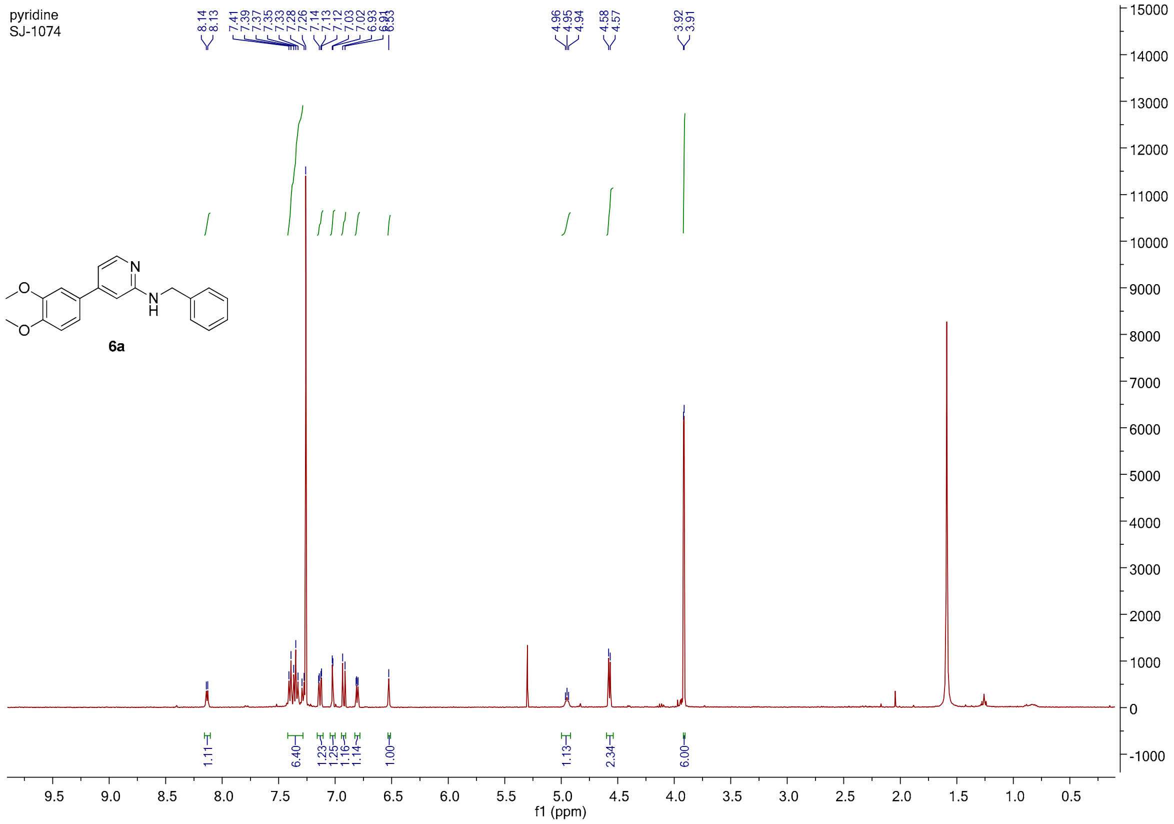


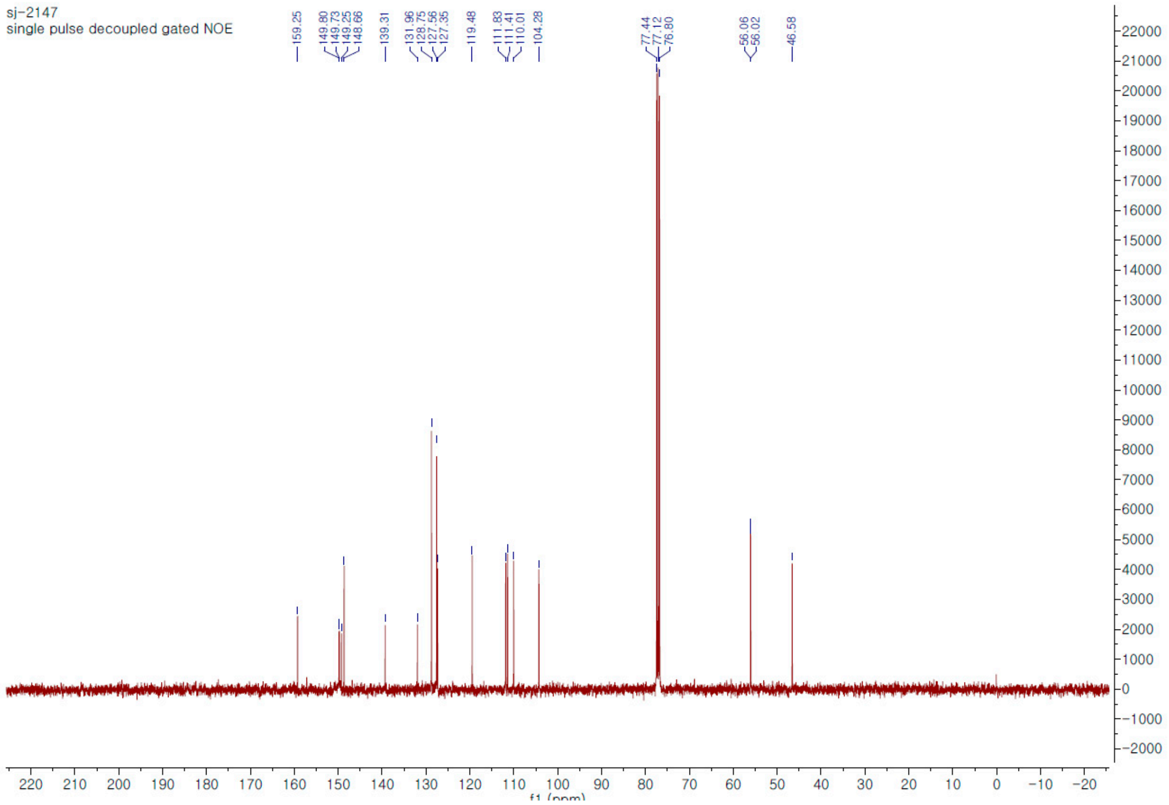

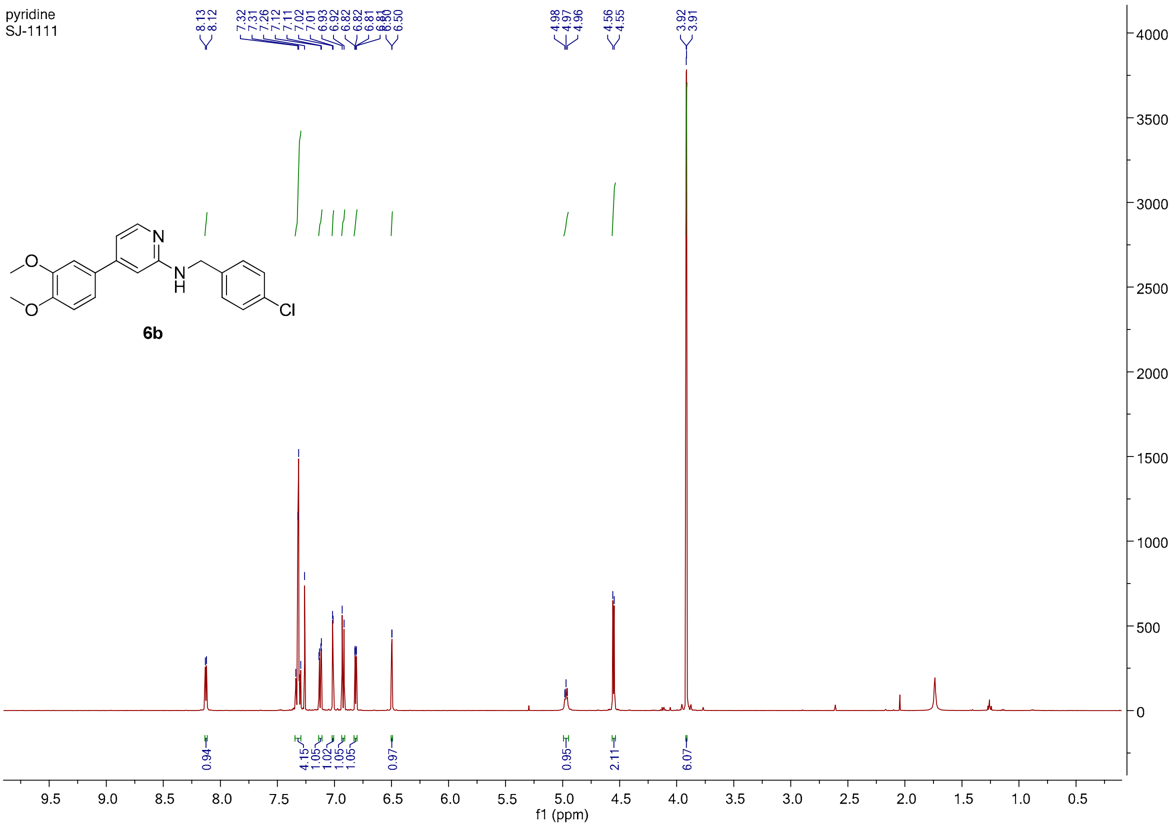
pyridine
SJ-1111
8.13 8.12
7.32 7.31 7.26 7.12 7.11 7.02 7.01 6.93 6.92 6.82 6.82 6.81 6.81 6.80 6.50
4.98 4.97 4.96
4.56 4.55
3.92 3.91
N
N
H
O
O
Cl
6b
0.94
4.15 1.05 1.02 1.05 1.05
0.97
0.95
2.11
6.07
f1 (ppm)

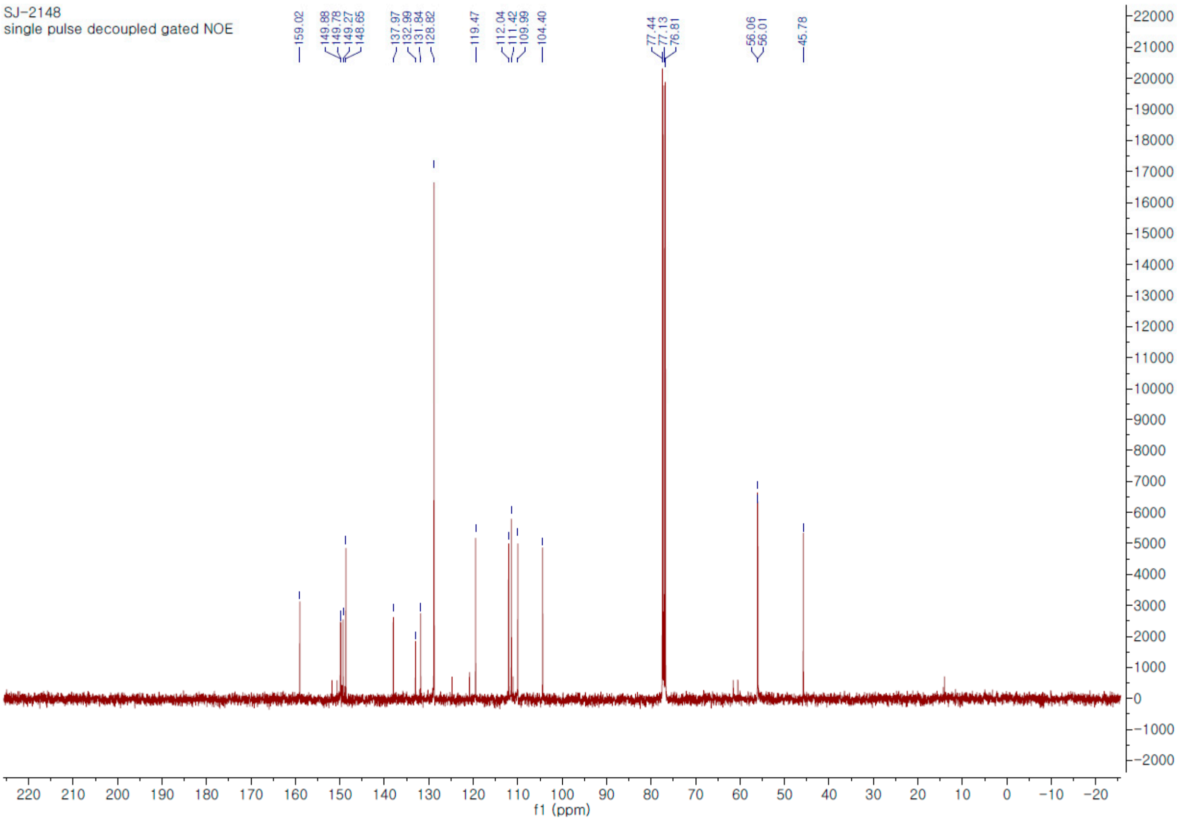
SJ-2148
single pulse decoupled gated NOE
159.02
149.88 149.78 149.27 148.65
137.97 132.99 131.84 128.82
119.47
112.04 111.42 109.99
104.40
77.44 77.13 76.81
56.06 56.01
45.78
f1 (ppm)

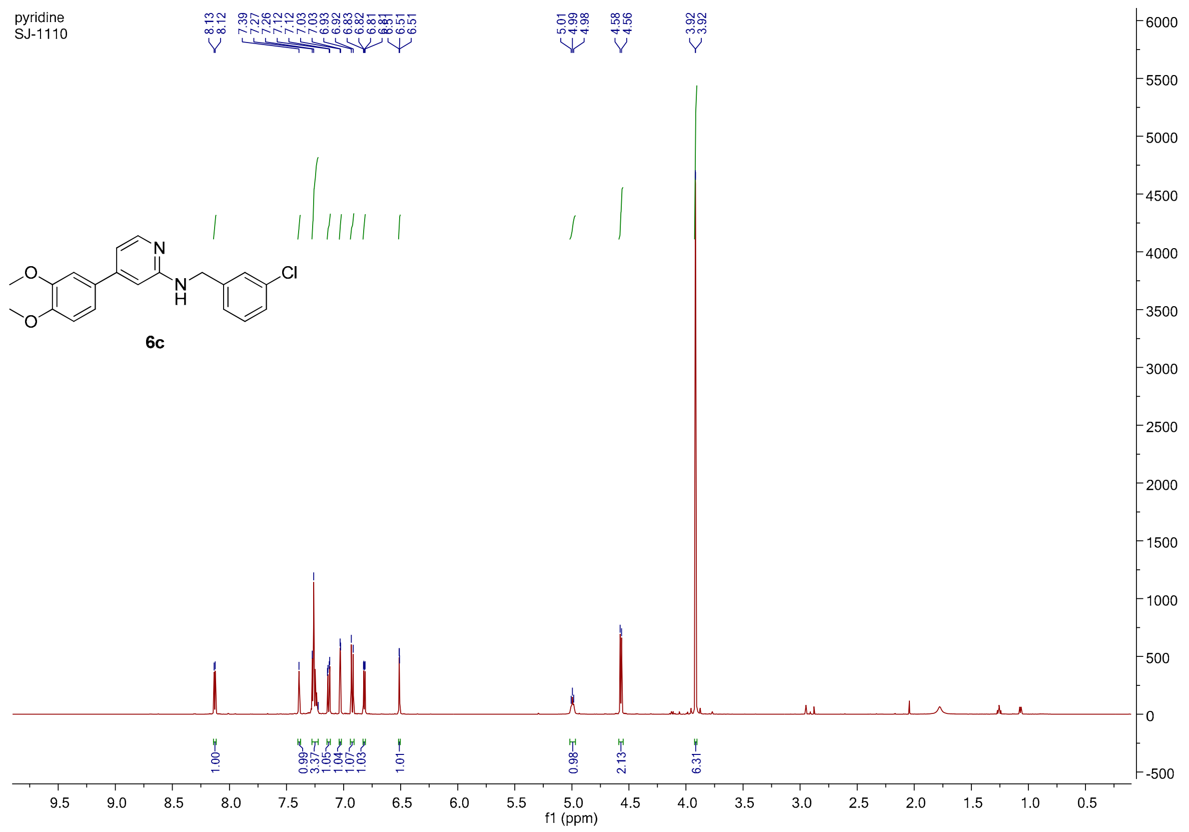
pyridine
SJ-1110
6c
f1 (ppm)

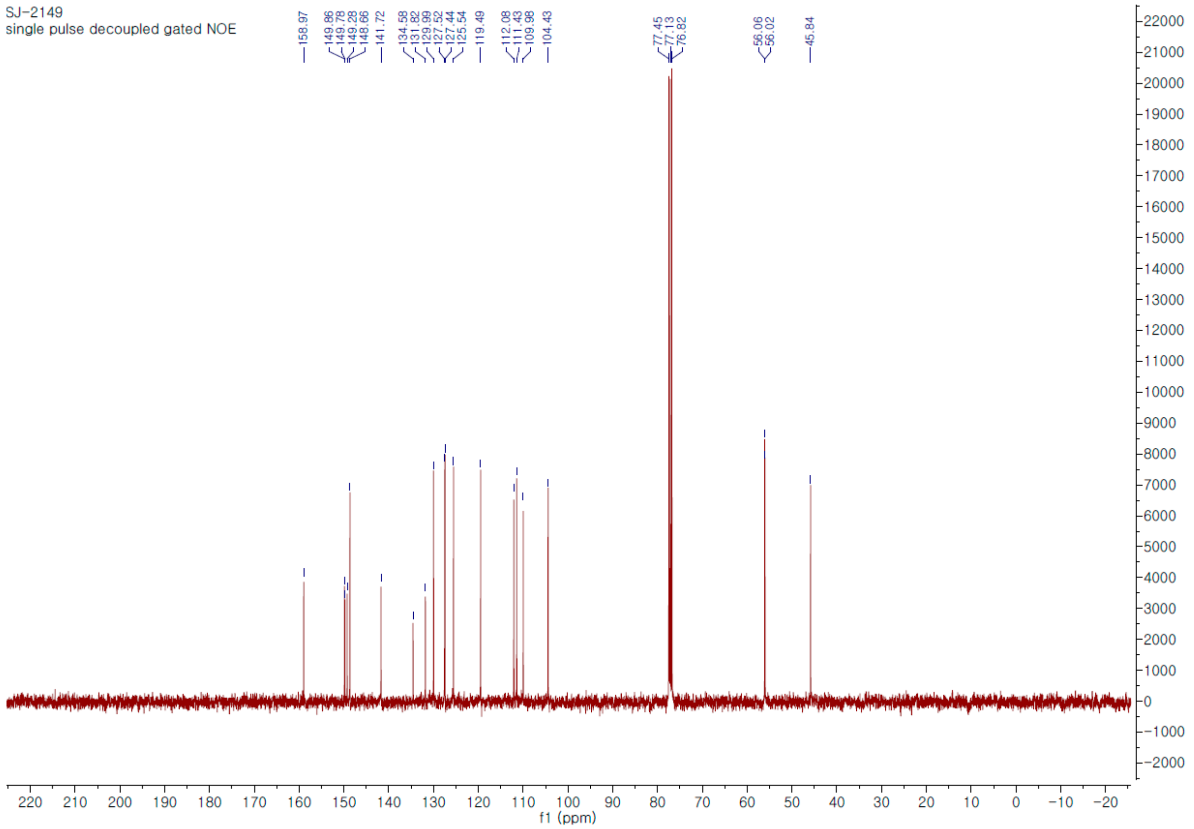
SJ-2149
single pulse decoupled gated NOE
f1 (ppm)

pyridine
SJ-1085

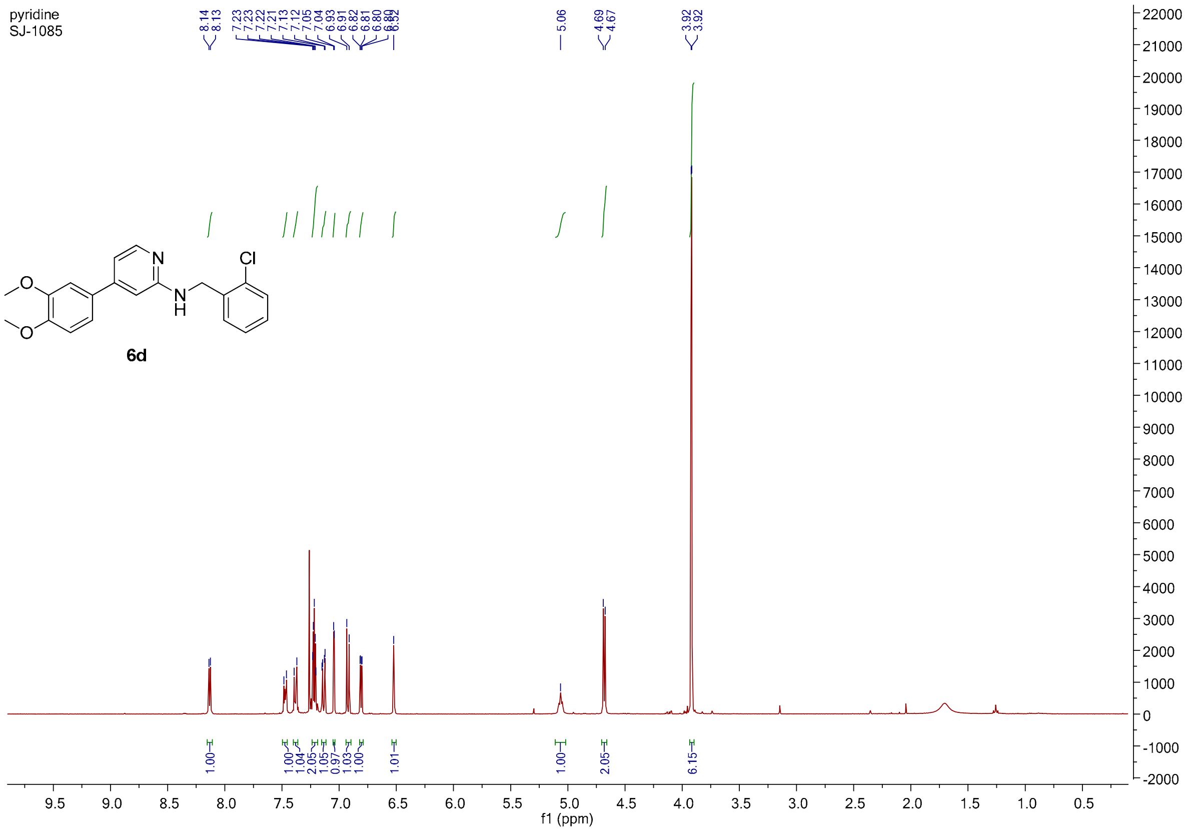

SJ-2150
single pulse decoupled gated NOE

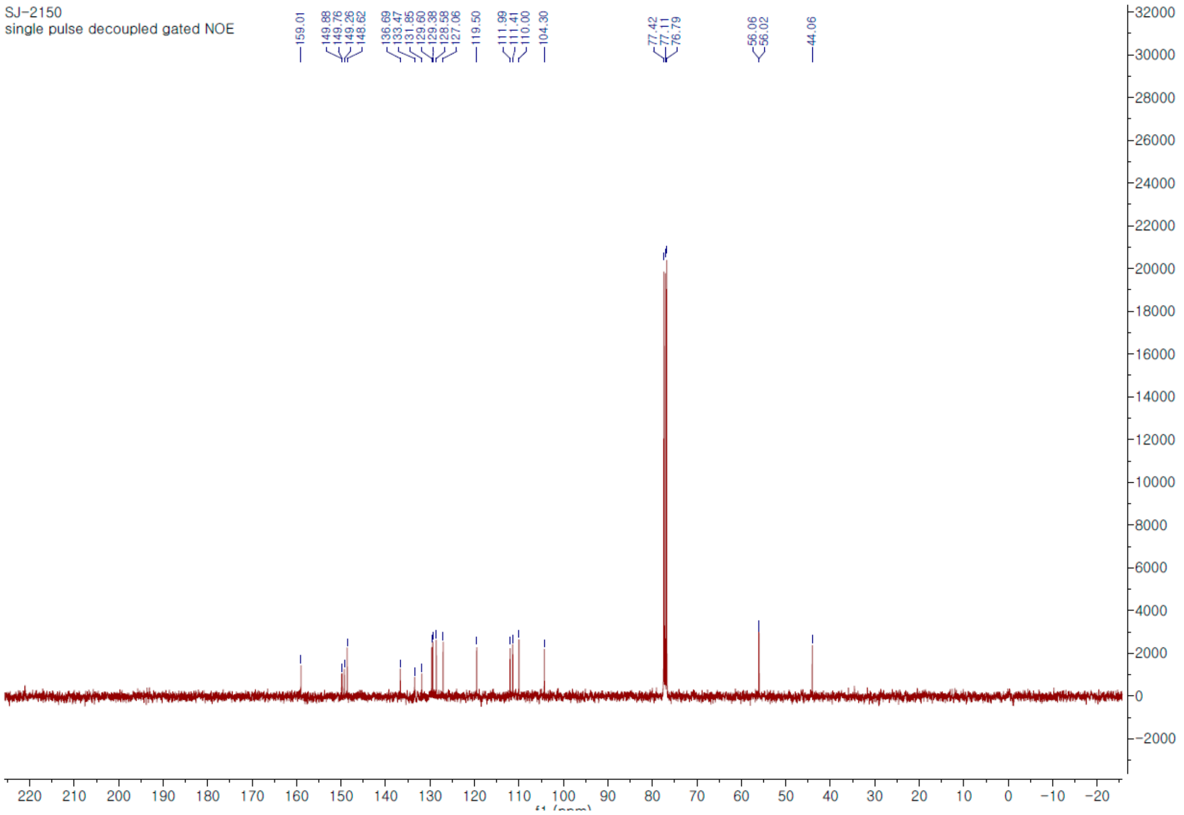

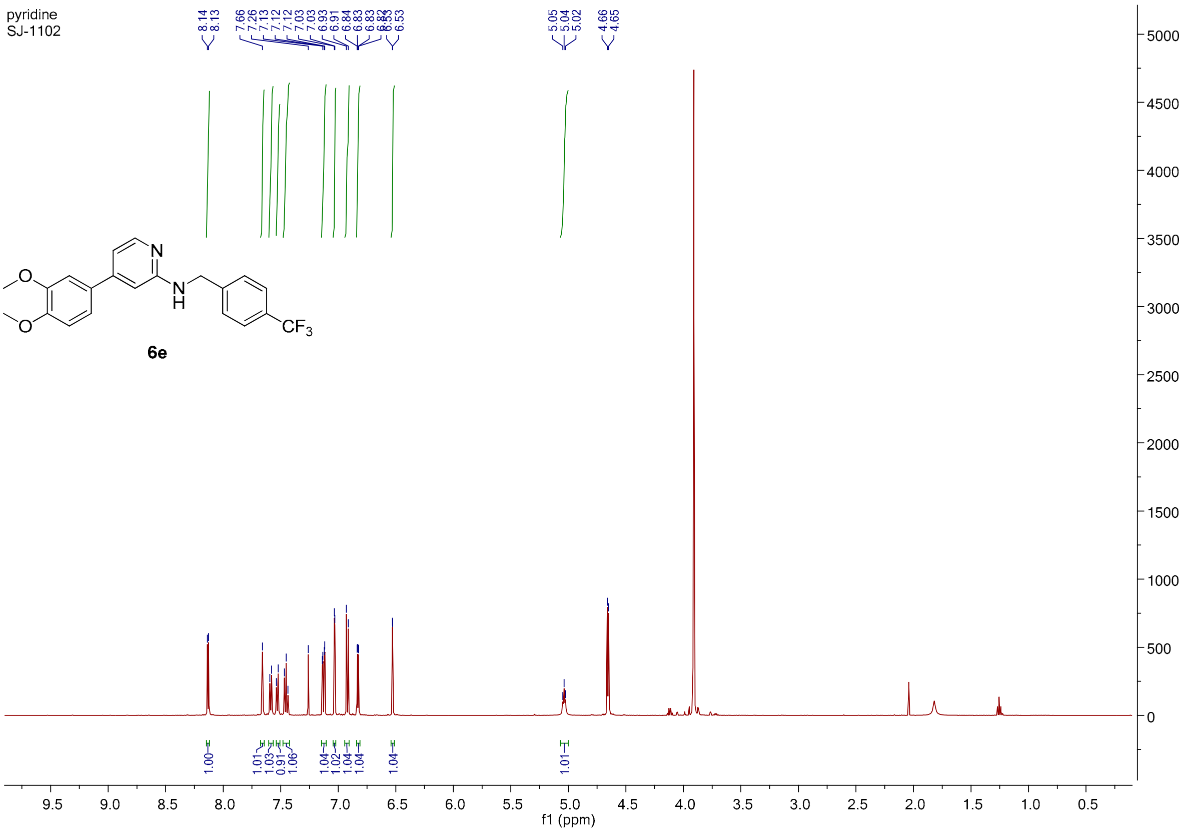

pyridine
SJ-1102
8.14
8.13
7.66
7.26
7.13
7.12
7.12
7.03
7.03
6.93
6.91
6.84
6.83
6.83
6.53
6.53
5.05
5.04
5.02
4.66
4.65
N
N
H
O
O
CF3
6e
1.00
1.01
1.03
0.91
1.06
1.04
1.02
1.04
1.04
1.04
1.01
f1 (ppm)


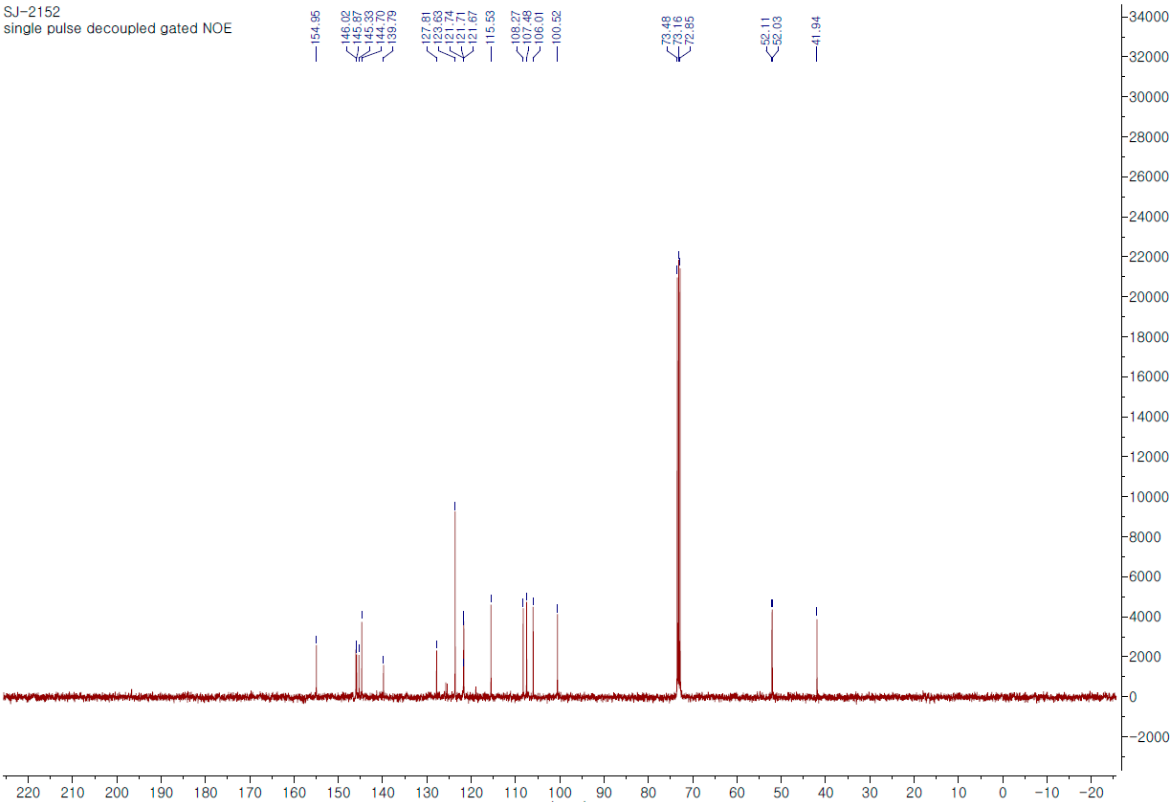

SJ-2152
single pulse decoupled gated NOE
154.95
146.02
145.87
145.33
144.70
139.79
127.81
123.63
121.74
121.71
121.67
115.53
108.27
107.48
106.01
100.52
73.48
73.16
72.85
52.11
52.03
41.94

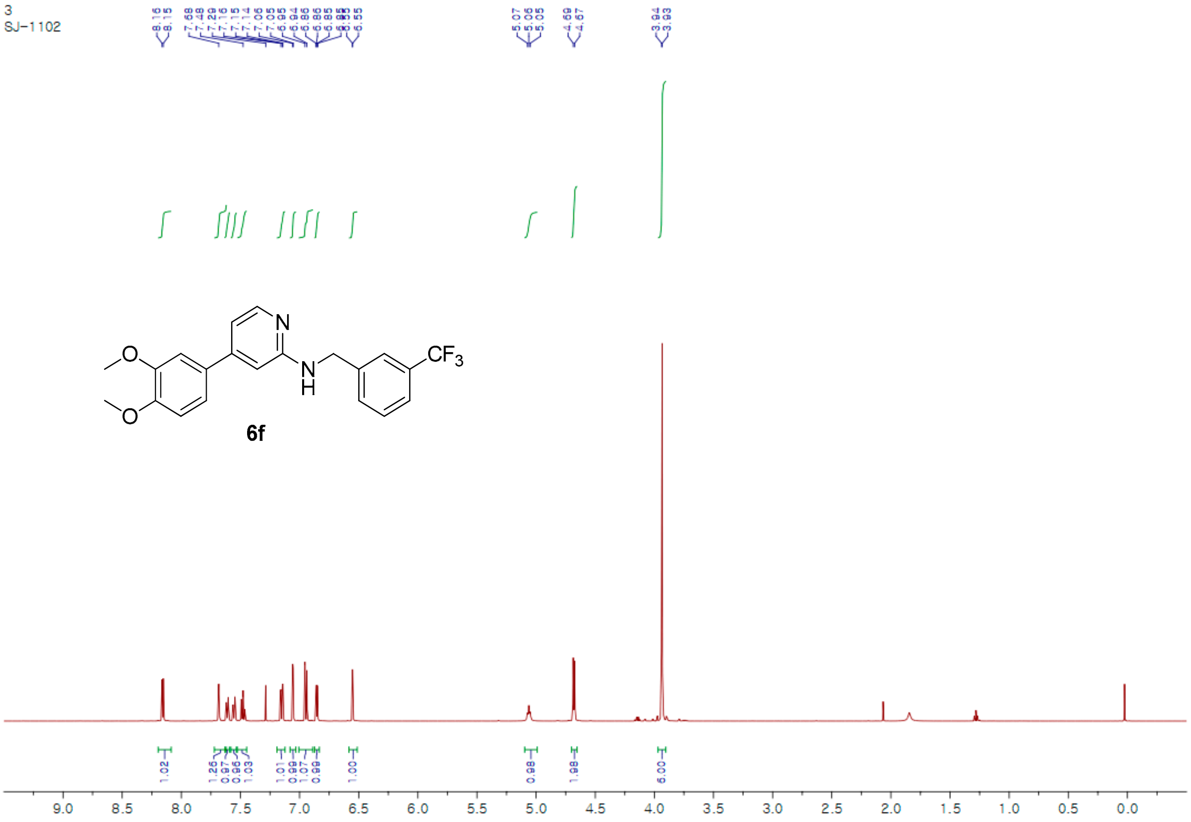
3
SJ-1102
6f
N
H
O
O
$CF_3$
8.16
8.15
5.07
5.06
5.05
4.69
4.67
3.94
3.93
1.02
0.98
1.98
6.00
9.0
8.5
8.0
7.5
7.0
6.5
6.0
5.5
5.0
4.5
4.0
3.5
3.0
2.5
2.0
1.5
1.0
0.5
0.0

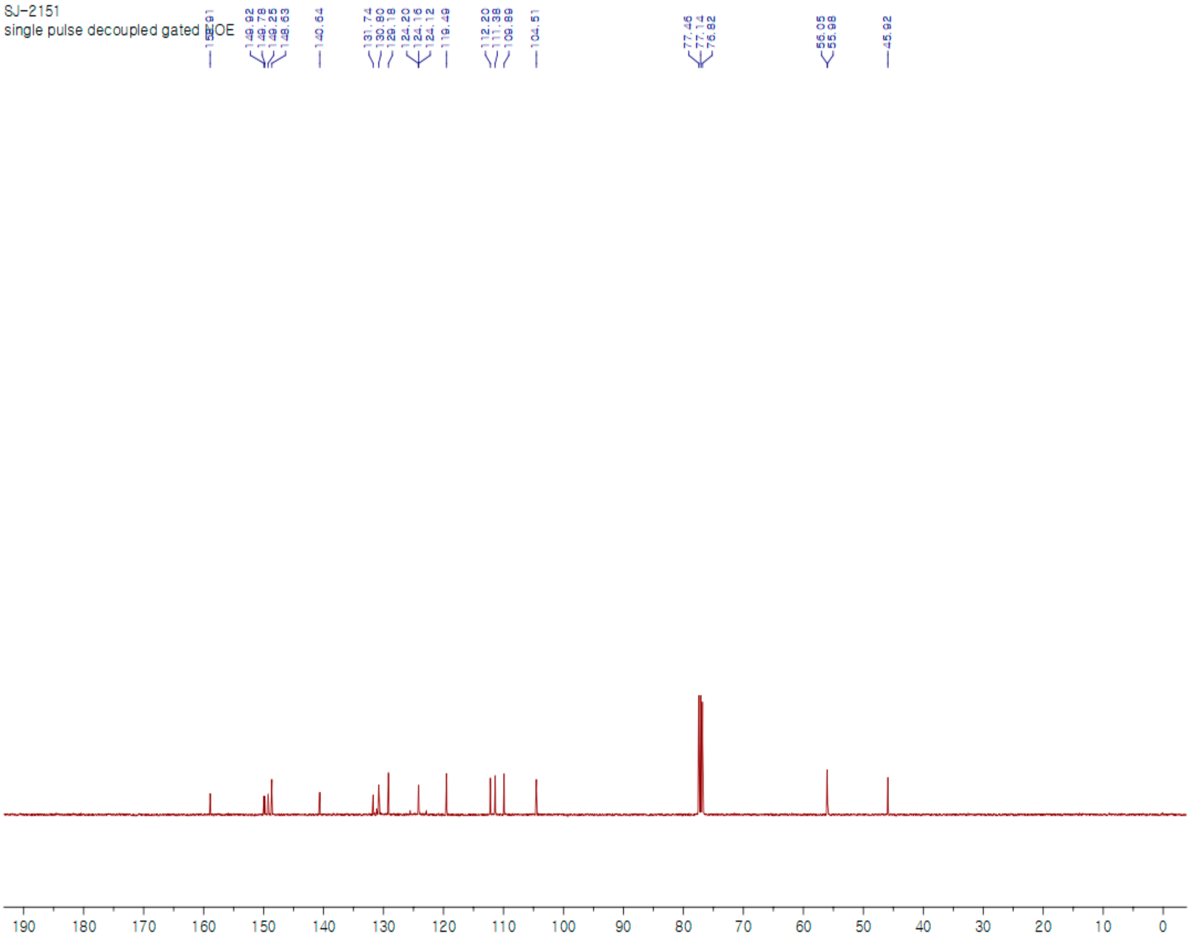
SJ-2151
single pulse decoupled gated NOE
149.92
149.78
149.25
148.63
140.64
131.74
130.80
129.18
124.20
124.16
124.12
119.49
112.20
111.38
109.89
104.51
77.46
77.14
76.82
56.05
55.98
45.92
190
180
170
160
150
140
130
120
110
100
90
80
70
60
50
40
30
20
10
0

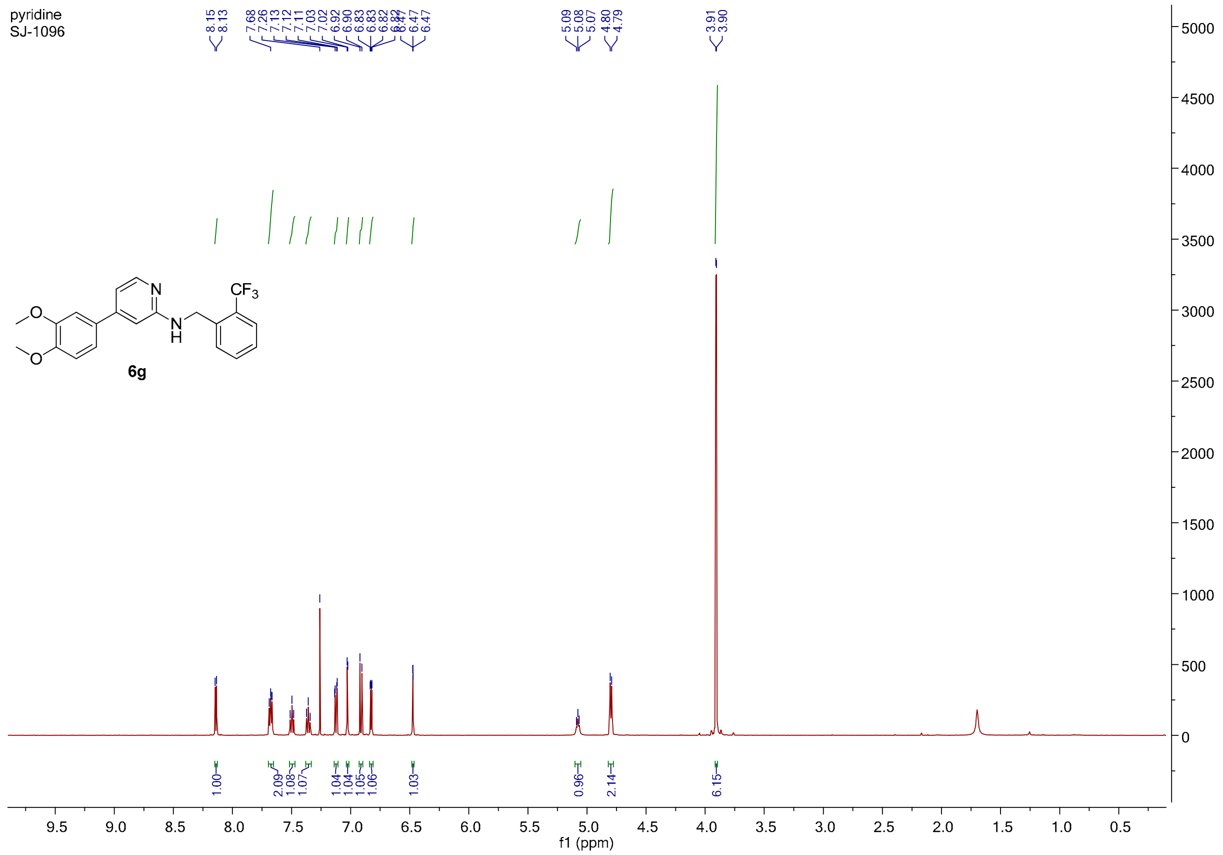
pyridine
SJ-1096
8.15
8.13
7.68
7.26
7.13
7.12
7.11
7.03
7.02
6.92
6.90
6.83
6.83
6.82
6.47
6.47
5.09
5.08
5.07
4.80
4.79
3.91
3.90
$CF_3$
O
O
N
N
H
6g
1.00
2.09
1.08
1.07
1.04
1.04
1.05
1.06
1.03
0.96
2.14
6.15
f1 (ppm)

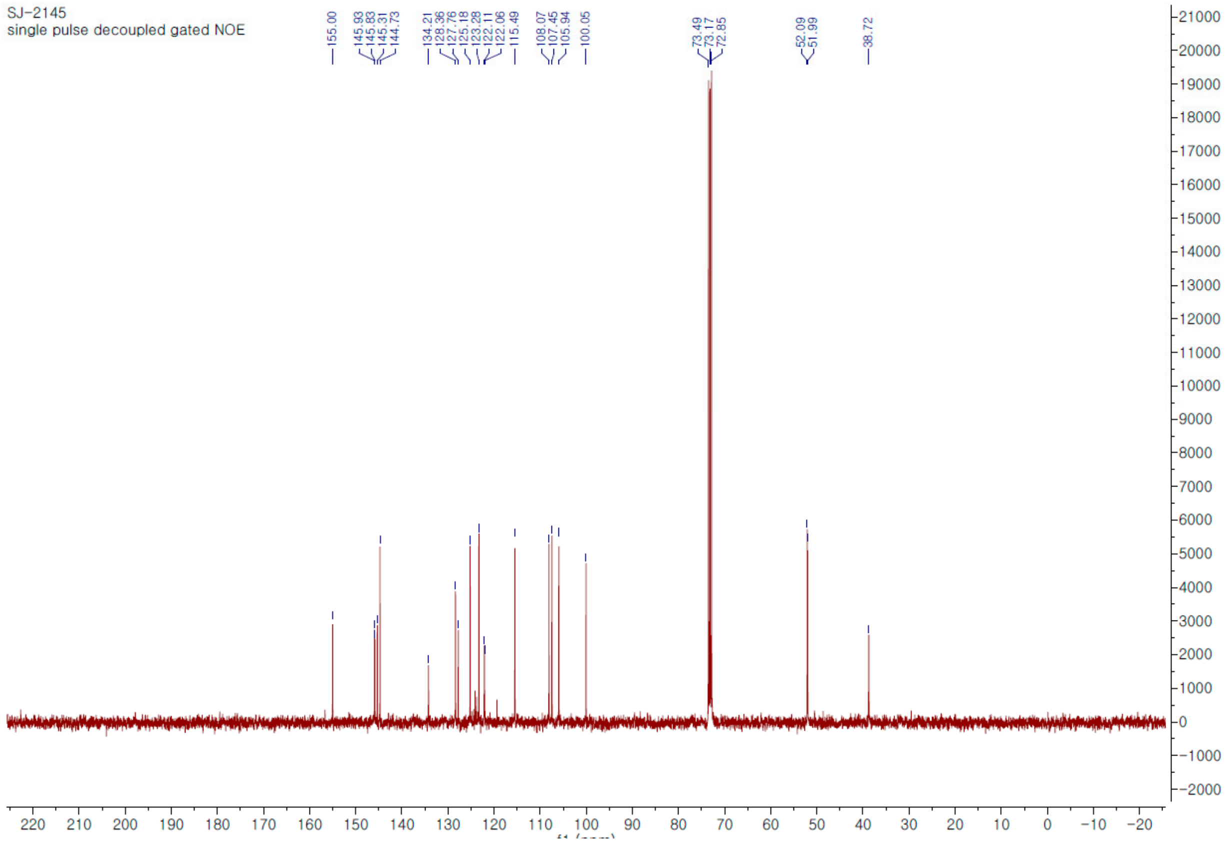
SJ-2145
single pulse decoupled gated NOE
155.00
145.93
145.83
145.31
144.73
134.21
128.36
127.76
125.18
123.28
122.11
122.06
115.49
108.07
107.45
105.94
100.05
73.49
73.17
72.85
52.09
51.99
38.72

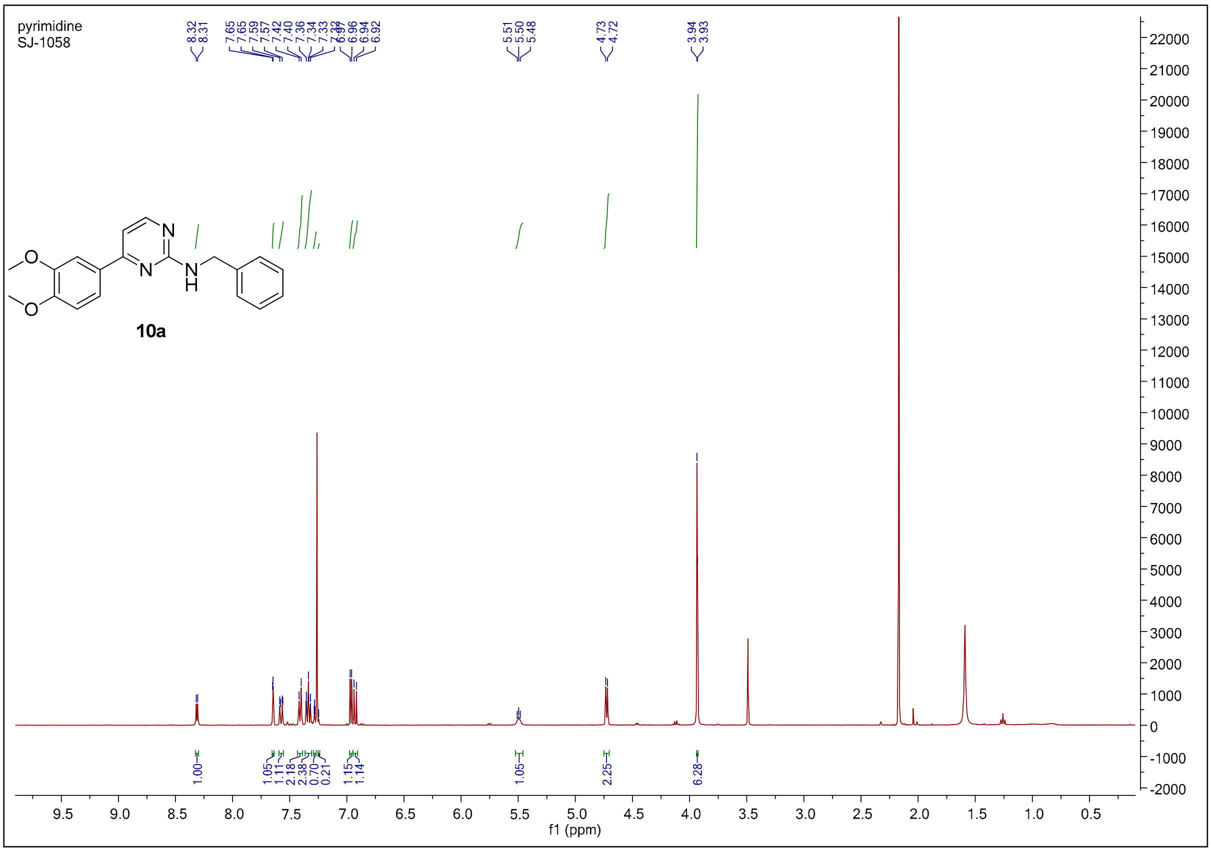
pyrimidine
SJ-1058
8.32
8.31
7.65
7.65
7.59
7.57
7.42
7.40
7.36
7.34
7.33
6.97
6.96
6.94
6.92
5.51
5.50
5.48
4.73
4.72
3.94
3.93
O
O
N
N
N
H
10a
1.00
1.05
1.11
2.18
2.38
0.70
0.21
1.15
1.14
1.05
2.25
6.28
f1 (ppm)

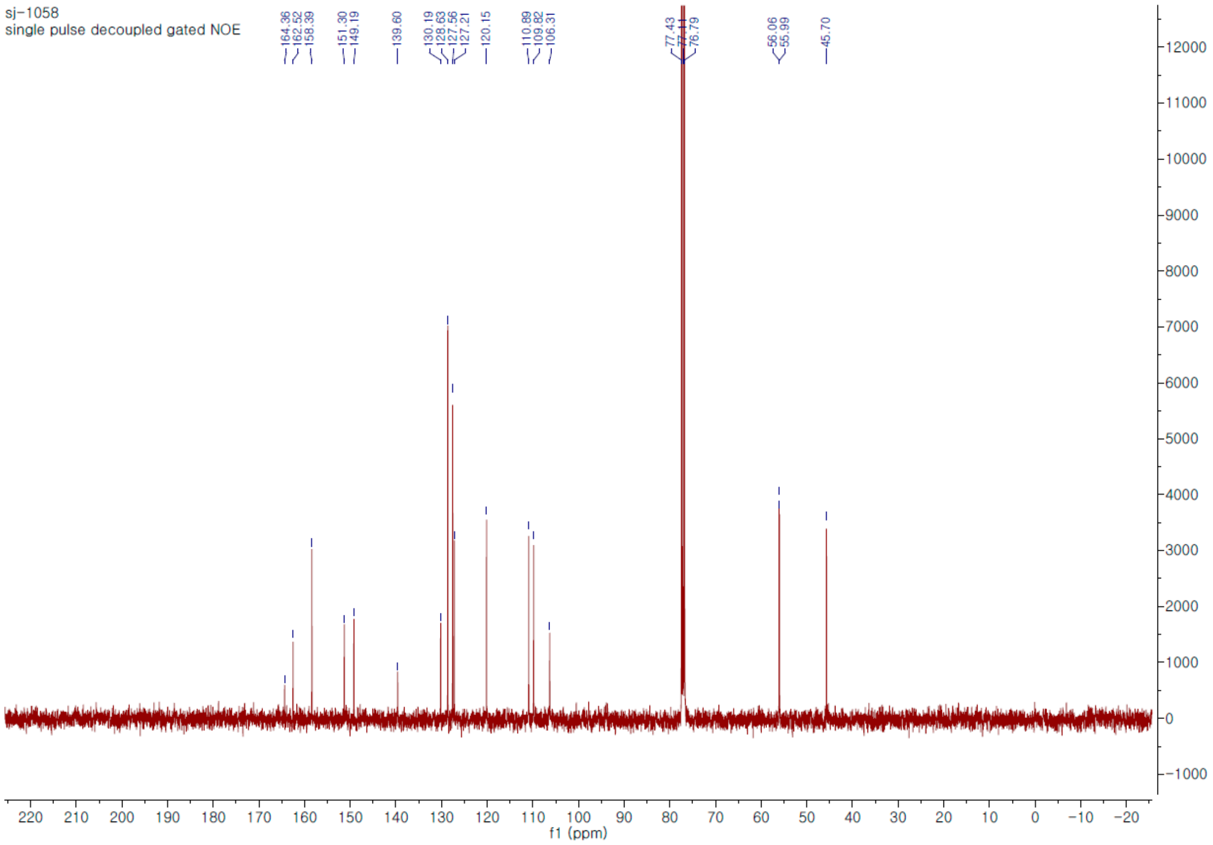
sj-1058
single pulse decoupled gated NOE
164.36
162.52
158.39
151.30
149.19
139.60
130.19
128.63
127.56
127.21
120.15
110.89
109.82
106.31
77.43
76.79
56.06
55.99
45.70
f1 (ppm)

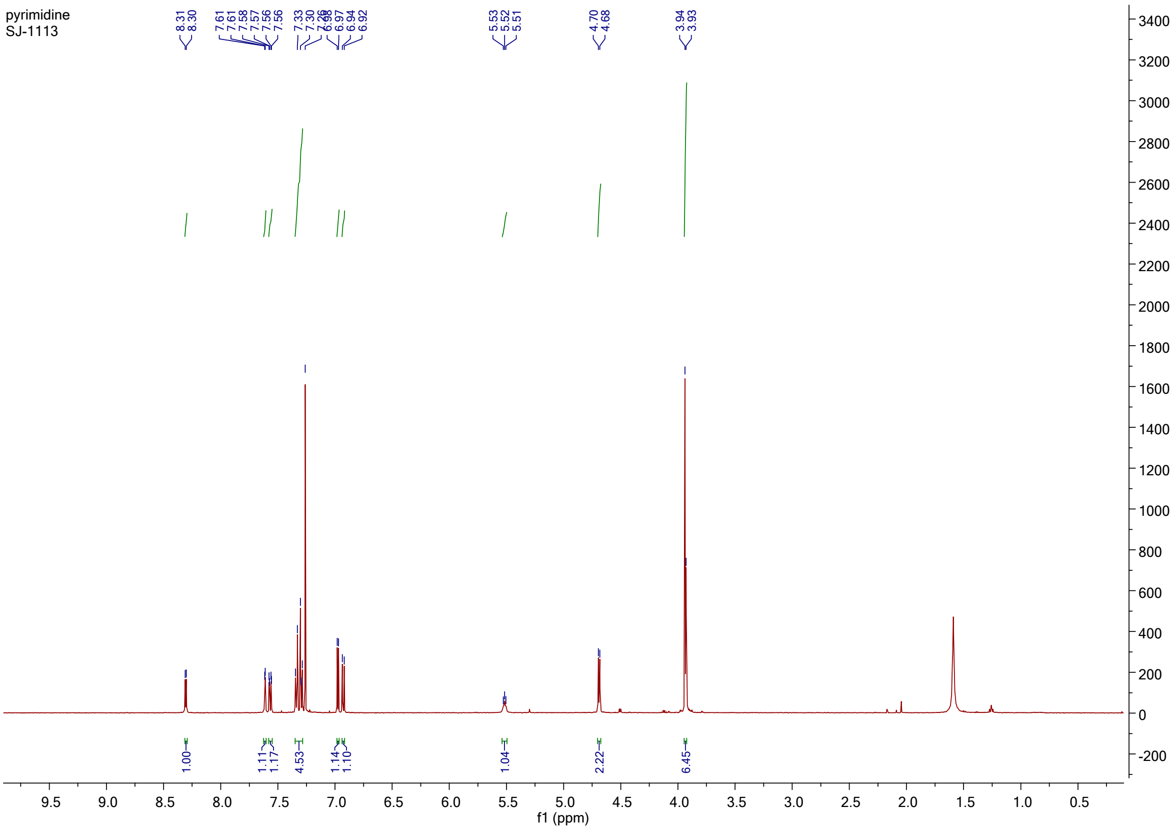
pyrimidine
SJ-1113
8.31
8.30
7.61
7.61
7.58
7.57
7.56
7.56
7.33
7.30
7.26
6.98
6.97
6.94
6.92
5.53
5.52
5.51
4.70
4.68
3.94
3.93
1.00
1.11
1.17
4.53
1.14
1.10
1.04
2.22
6.45
f1 (ppm)
